# Retinoic acid-stimulated ERK1/2 pathway regulates meiotic initiation in cultured fetal germ cells

**DOI:** 10.1371/journal.pone.0224628

**Published:** 2019-11-04

**Authors:** Sung-Min Kim, Toshifumi Yokoyama, Dylan Ng, Ferhat Ulu, Yukiko Yamazaki

**Affiliations:** 1 Institute for Biogenesis Research, John A. Burns School of Medicine, University of Hawaii, Honolulu, HI, United States of America; 2 Department of Animal Science, Kobe University, Kobe, Hyogo, Japan; Duke University School of Medicine, UNITED STATES

## Abstract

In murine fetal germ cells, retinoic acid (RA) is an extrinsic cue for meiotic initiation that stimulates transcriptional activation of the *Stimulated by retinoic acid gene 8* (*Stra8*), which is required for entry of germ cells into meiotic prophase I. Canonically, the biological activities of RA are mediated by nuclear RA receptors. Recent studies in somatic cells found that RA noncanonically stimulates intracellular signal transduction pathways to regulate multiple cellular processes. In this study, using a germ cell culture system, we investigated (1) whether RA treatment activates any mitogen-activated protein kinase (MAPK) pathways in fetal germ cells at the time of sex differentiation, and (2) if this is the case, whether the corresponding RA-stimulated signaling pathway regulates *Stra8* expression in fetal germ cells and their entry into meiosis. When XX germ cells at embryonic day (E) 12.5 were cultured with RA, the extracellular-signal-regulated kinase (ERK) 1/2 pathway was predominantly activated. MEK1/2 inhibitor (U0126) treatment suppressed the mRNA expressions of RA-induced *Stra8* and meiotic marker genes (*Rec8*, *Spo11*, *Dmc1*, *and Sycp3*) in both XX and XY fetal germ cells. Furthermore, U0126 treatment dramatically reduced STRA8 protein levels and numbers of meiotic cells among cultured XX and XY fetal germ cells even in the presence of RA. Taken together, our results suggest the novel concept that the RA functions by stimulating the ERK1/2 pathway and that this activity is critical for *Stra8* expression and meiotic progression in fetal germ cells.

## Introduction

Primordial germ cells (PGCs) are the embryonic precursors of oogonia and prospermatogonia in mammals. In mouse fetuses, early-stage PGCs continue to proliferate mitotically and migrate through the somatic tissues to eventually colonize the gonads at approximately embryonic day (E) 10.5. In the gonads, fetal germ cells are induced to undergo sex differentiation depending on the somatic gonadal environment rather than on their sex chromosome constitution. A common feature of female-specific sex differentiation in developing ovaries is entry into meiosis. Thus, in an ovarian environment, XX germ cells immediately enter meiotic prophase I at E12.5–13.5 and proceed to the diplotene stage by E17.5 [1–3]. However, in a testicular environment, XY germ cells at E13.5–15.5 are blocked from initiating meiosis. Thus, XY germ cells give rise to M prospermatogonia, which continues to expand mitotically before entering a mitotically quiescent G0/G1 stage of the cell cycle as T1 prospermatogonia [3,4]. After birth, male germ cells resume mitotic proliferation as T2 prospermatogonia and spermatogonia, and subsequently initiate meiosis at about 8–10 days postpartum (dpp) [3–5].

Although the timing of meiotic entry exhibits distinct differences during oogenesis and spermatogenesis, all-*trans* retinoic acid (RA) has been widely recognized as a key regulator of entry into meiosis in both male and female germ cells [6–10]. Multiple studies have shown that RA treatment can induce PGCs in E11.5 male genital ridges or isolated XY germ cells at E12.5–14.5 to initiate entry into meiosis [6,7,11–14]. In germ cells of either sex, RA stimulates the expression of *Stimulated by retinoic acid (Stra8)*, a meiotic “gatekeeper” gene by controlling the switch from mitosis to meiosis [7–9,15]. Studies with a *Stra8*-defecient mouse model demonstrated that *Stra8* is required for premeiotic DNA replication and the subsequent events of meiotic prophase in female germ cells [15,16]. In XX germ cells, *Stra8* is expressed at E12.5 before these cells enter meiotic prophase I [17]. Previous studies have shown a clear connection between RA and *Stra8* expression in fetal germ cells [6,7,13,18]; however, although multiple lines of evidence reinforce the importance of the molecular connection between RA and *Stra8*, it remains unclear how RA regulates *Stra8* expression in germ cells.

In mammalian cells, RA plays multiple key roles in proliferation [19,20], apoptosis [21] and differentiation [22]. Canonically, the biological activity of RA signaling is mediated by nuclear RA receptors (RAR α, β and γ) [23]. In the cytoplasm, RA molecules bind to cellular RA-binding proteins (CRABPs), move to the nucleus, and directly bind to RARs. Nuclear RARs form heterodimers with retinoid X receptors (RXR α, β and γ) to function as ligand-dependent transcriptional regulators. The RAR/RXR heterodimers interact with RA response elements (RAREs) localized in the promoter regions of RA target genes, which, in turn, activates their transcription [24]. This classical RA-signaling mechanism represents the canonical activity of RA.

*Stra8* is one of the RA target genes, and its promoter region contains putative RAREs, [9,25–27]. Using a chromatin immunoprecipitation (ChIP) assay, it has been shown that RARs directly bind to the RAREs at the *Stra8* promoter region in the postnatal mouse testis [9]. Furthermore, numerous pharmacological approaches have shown successful inhibition of *Stra8* expression by RAR antagonists, resulting in subsequent inhibition of initiation of meiosis in fetal germ cells [6,7,11]. These previous studies suggest that the transcriptional expression of RA-induced *Stra8* is controlled by the canonical activity of RA mediated via nuclear RARs in germ cells.

Recent studies have reported that RA signaling is also mediated by intracellular signal transduction pathways that do not rely upon binding to the nuclear RARs (noncanonical activity of RA) [28, 29]. In the cytoplasm, RA stimulates signal transduction pathways such as the mitogen-activated protein kinase (MAPK) [29], protein kinase C (PKC) [30] and AKT pathways [31]. Particularly, the MAPK pathways, including the extracellular signal-regulated kinase (ERK) 1/2 and p38 MAPK, are major targets of the noncanonical activity of RA [28,29]. These studies have demonstrated that the noncanonical activity of RA appears to contribute to different mechanisms and kinase cascades in different types of somatic cells [20–22,32–39]. Importantly, the activation of these multiple kinase cascades contributes to the transcriptional activity of RA target genes [37]. In germ cells, recent studies have also suggested that RA-induced meiotic initiation is mediated by intracellular signal transduction pathways [40, 41]. Pellegrini et al. (2008) reported that RA activates Kit phosphorylation and its downstream signaling pathways, PI3K and MAPK, to induce meiotic entry of postnatal mouse spermatogonia [40]. It was also suggested that JNK signaling contributes to meiosis associated gene expression [41]. However, it is still unclear if the biological activity of RA is mediated by any specific signal transduction pathway controlling *Stra8* expression in fetal germ cells. In this study, using a germ cell culture system, we investigated (1) whether RA activates any MAPK pathways (ERK1/2, p38 MAPK and JNK) in fetal germ cells, and (2) if this is the case, whether the corresponding RA-stimulated signal pathway(s) regulate(s) *Stra8* expression and following entry into meiosis in fetal germ cells. Our results indicate that RA predominantly activates the ERK1/2 pathway to control *Stra8* expression and meiotic initiation in fetal germ cells in vitro.

## Materials and methods

### Mice

Mice carrying a *Pou5f1*-green fluorescent protein (GFP) transgene (Tg OG2) were generated by microinjecting (CBA/Caj X C57BL/6J) F_2_ zygotes and expressing germ cell-specific GFP driven by the *pou5fl* gene promoter/enhancer (a generous gift from Dr. Jeff R. Mann, University of Melbourne, Melbourne, VIC, Australia) [42]. CD-1 female mice (Charles River, San Diego, CA, USA) were mated with Tg OG2 homozygous males to produce (CD-1 X OG2) F_1_ hybrid fetuses. For all experiments, GFP-positive germ cells were isolated from these F1 fetuses (see below). All experiments involving mice were reviewed and approved in advance by the Institutional Animal Care and Use Committee of the University of Hawaii and all procedures were in accordance with the guidelines recommended by the National Institute of Health.

### Media

Gonads were dissected in Hepes-Dulbecco’s Modified Eagle Medium (DMEM; Invitrogen, Carlsbad, CA, USA) supplemented with 15% fetal bovine serum (FBS; Hyclone Laboratories, Logan, UT, USA). For germ cell culture, sorted germ cells were cultured in a basic medium (high-glucose DMEM supplemented with 0.1 mM nonessential amino acids, 0.1 mM 2-merkaptoethanol, 100 IU/ml penicillin, 100 μg/ml streptomycin, 2 mM glutamine and 1 mM sodium pyruvate) supplemented with 15% FBS, with or without 1 μM all-*trans* RA (Sigma-Aldrich, St. Louis, MO, USA) and/or MEK1/2 inhibitor (U0126; Cayman Chemical Company, Ann Arbor, MI, USA) at 10, 20 or 50 μM. For gonadal culture experiments, the tissues were cultured with a basic medium with or without 50 μM MEK1/2 inhibitor (U0126).

### Culture of fetal germ cells

XY and XX germ cells were freshly isolated from male or female gonads, respectively, at E11.5, 12.5, 13.5, 14.5 and 15.5, as described previously [43]. The sexes of E11.5 fetuses were determined by germ cell amplification of genomic DNA for the *Sry* gene, using specific primers (5’-CTGTGTAGGATCTTCAATCTCT-3’ and 5’-GTGGTGAGAGGCACAAGTTGGC-3’) [44]. Briefly, male and female gonads were dissected in Hepes-DMEM supplemented with 15% FBS and incubated in 0.2% Collagenase (Calbiochem, San Diego, CA, USA) and Accumax (Innovative Cell Technologies, San Diego, CA, USA) for 10 min each at 37°C. After enzymatic treatment, gonads were dissociated with pipetting and filtered through a 30 μm filter (Sysmex America, Lincolnshire, IL, USA) to prepare a single cell suspension. GFP-positive cells were sorted using a FACS Aria system (BD Bioscience, San Jose, CA, USA). After sorting, the purity of GFP-positive cells (the ratio of GFP-positive cells in total cells) was ≥ 95%. Isolated cells were either used as a fresh sample (no culture) or placed on collagen-coated mesh inserts (Corning Life Science, Lowell, MA, USA) to culture for 0.5–72h with or without RA and/or U0126. These cells were subjected to the quantitative polymerase chain reaction (qPCR), Western blot, and immunostaining analyses.

### Culture of female gonads

Female gonads with the mesonephros (gonad + meso) were dissected from the E12.5 fetuses. One gonad + meso per pair was used in the control group and the other was used in the 50 μM MEK inhibitor (U0126) treatment group. The gonad + meso tissues (2–4 tissues as one group) were placed on a 2% agar block soaked in the basic medium (control) or in the basic medium with the inhibitor (treatment). After 24 or 48h of culture, the gonads were dissected from the mesonephros and subjected to the enzymatic treatment as shown above. After the treatment, gonads were dissociated with pipetting to make single-cell suspensions. GFP-positive germ cells in the cell suspension were visualized under epi-illumination and morphologically healthy cells with a large nucleus and smooth cytoplasm were manually collected (200 cells per set) by using a glass pipette fitted to an inverted microscope (200x) (Olympus IX71, Center Valley, PA, USA) as described previously [45]. The collected germ cells were subjected to the quantitative gene expression analysis.

### Quantitative gene expression analysis

After culturing the germ cells for 24-72h, morphologically healthy germ cells with a large nucleus and smooth cytoplasm were collected manually (200–300 cells per set) with a glass pipette using an inverted microscope (200x) (Olympus IX71). After the gonadal culture, 200 GFP-positive cells per set were isolated for the analysis. The collected germ cells were subjected to cDNA synthesis using the SuperScript III Cells Direct cDNA synthesis kit (Invitrogen). qPCR analysis was performed using a MyiQ Single-Color Real-Time PCR Detection system (Bio-Rad Laboratories, Hercules, CA, USA). Each experiment was repeated three to five times to provide biological replicates, with duplicate samples of each biological replicate were assayed to provide technical replicates. As previously describe [43], the sequences of primers used were as follows (forward and reverse, respectively): for *Stra8*, 5’–GTTTCCTGCGTGTTCCACAAG–3’ and 5’–CACCCGAGGCTCAAGCTTC–3’; for *Rec8*, 5’–CTACCTAGCTTGCTTCTTCC–3’ and 5’–GCCTCTAAAAGGTGTCGAA–3’; for *DNA meiotic recombinase 1* (*Dmc1*), 5’–CCCTCTGTGTGACAGCTCAAC–3’ and 5’–GGTCAGCAATGTCCCGAAG–3’; for *Synaptonemal complex protein 3* (*Sycp3*), 5’–GTGCCTGGTGGAAGAAAGCA–3’ and 5’–GGAGCCTTTTCATCAGCAACAT–3’; for *Spo11*, 5’–CGTGGCCTCTAGTTCTGAGGT–3’ and 5’–GCTCGATCTGTTGTCTATTGTGA–3’; for *DNA methyltransferase 3-like* (*Dnmt3L)*, 5’–GTGCGGGTACTGAGCCTTTTTAGA–3’ and 5’–CGACATTTGTGACATCTTCCACGTA–3’; for *β-actin*, 5’–CCTGTATGCCTCTGGTCGTA–3’ and 5’–CCATCTCCTGCTCGAAGTCT–3’; for *Mouse Vasa homolog (Mvh)*, 5’–CTCAAACAGGGTCTGGGAAG–3’ and 5’–TGGTTGATCAGTTCTCGAGT–3’. The results were normalized to levels detected for *β-actin* gene expression and the levels of transcripts were presented as expression levels relative to control groups, with the latter arbitrarily set as 1.00 as described previously [13,43,46]. The qPCR analysis was repeated independently in triplicate for each experiment.

### Western blotting

For Western blot analysis, cell lysates were prepared from 50,000 germ cells incubated for short periods (0.5-2h) or 24-48h with or without RA and/or U0126. Equal amounts of cell lysates were loaded into each lane of a 12% sodium dodecyl sulfate polyacrylamide gel electrophoresis (SDS-PAGE) gel for electrophoresis, and the contents of the subsequent gel were electrotransferred onto polyvinyl difluoride membranes (GE Healthcare, Chicago, IL, USA). The transferred membranes were blocked with 5% non-fat dried milk in 0.1% Tween 20 in phosphate-buffered saline (PBS-T), and then probed with primary antibodies (all purchased from Cell Signaling Technology, Danvers, MA, USA) (S1 Table) against β-ACTIN (1:1000), ERK1/2 (1:1000), phospho-ERK1/2 (1:1000), p38 (1:500), phospho-p38 (1:500), AKT (1:500), phospho-AKT (1:500), JNK (1:500), or phospho-JNK (1:500) overnight at 4°C. The following day, the membranes were rinsed 3x with PBS-T for 10 min each and incubated with an anti-rabbit immunoglobulin G (IgG), horseradish peroxidase-linked secondary antibody (1:1000; Cell Signaling Technology) (S1 Table) for 1h at room temperature. The membranes were exposed to X-ray film (GE Healthcare) to visualize the protein bands. The optical densities (O.D.) of the protein bands were quantified by densitometry using ImageJ software (https://imagej.nih.gov/ij/). For ERK1/2, P38, JNK and AKT, the total proteins and phospho-proteins were normalized to the *β*-ACTIN, and then the phospho/total protein ratios were determined. STRA8 proteins were normalized to the *β*-ACTIN. The fold changes of normalized phosphor/total and STRA8 proteins were calculated based on this comparison to control levels set as 1.0. The Western blotting analysis was repeated twice.

### Immunochemical staining

After 24 or 48h of culture, morphologically healthy germ cells (about 200–400 cells per set) were placed on glass slides and fixed with 4% paraformaldehyde (Sigma-Aldrich) for 15 min at room temperature. After washing, the cells were permeabilized with 0.2% Triton X-100 in PBS for 15 min, then blocked with 5% bovine serum albumin (BSA; Sigma-Aldrich) in PBS for 1h at room temperature. These cells were incubated with primary antibodies overnight at 4°C. Primary antibodies for immunostaining included (S1 Table): rabbit polyclonal anti-STRA8 (1:500; Abcam, Cambridge, MA, USA) and mouse polyclonal anti-γH2AX (1:500; Abcam). After washing, the cells were incubated with Alexa Fluor 568-conjugated goat anti-rabbit IgG (1:500; Thermo Fisher Scientific, Waltham, MA, USA) or Alexa Fluor 568-conjugated goat anti-mouse IgG (1:500; Thermo Fisher Scientific) (S1 Table) in 1% BSA in PBS, and subsequently counterstained with Hoechst 33342 (Invitrogen) for 15 min and mounted in 50% (v/v) glycerol in PBS. STRA8-positive and γH2AX-positive cells were detected and imaged using an Axio microscope (Carl Zeiss Microimaging, Göttingen, Germany). In the present study, the immunochemical staining was repeated independently in triplicate for each experiment.

### Statistical analysis

Data are represented as the mean ± standard errors of the means (SEM). For comparison of quantitative mRNA expression levels and the fluorescence intensity of STRA8 protein, data were analyzed by a one- or two-way analysis of variance (ANOVA) followed by Tukey’s multiple comparison test for normally distributed data. Chi-square test was used to compare categorical variables for the proportion of STRA8- and γH2AX-positive cells under different condition. A p-value < 0.05 was considered statistically significant. All statistical analyses were performed using GraphPad Prism software (4.0; GraphPad Software, San Diego, CA, USA).

## Results

### *Stra8* expression in endogenous XX and XY germ cells

**Fig 1:**
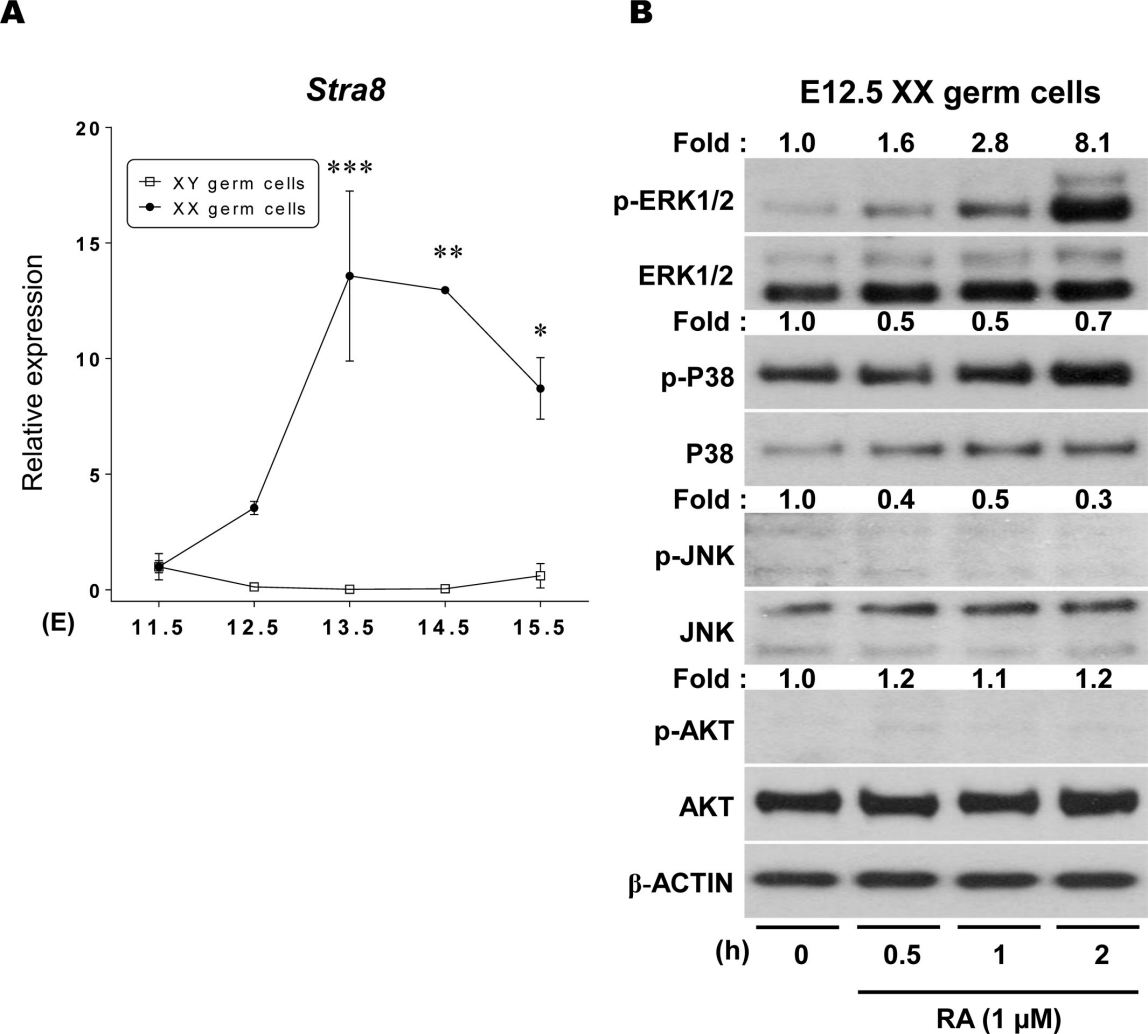
MAPK and AKT transduction pathways in XX germ cells cultured with RA. (A) The *Stra8* transcript levels were analyzed in freshly isolated XX and XY germ cells at E11.5 ~ 15.5. Results were normalized to the *β-actin* mRNA expression. All expression values were calculated relative to control levels set at 1.0. Data represent the mean ± SEM (n = 3). * p < 0.05, ** p < 0.01, *** p < 0.001 *vs*. E11.5. (B) To determine whether RA activates MAPK (ERK1/2, p38 MAPK and JNK) and AKT pathways, isolated XX germ cells at E12.5 were incubated with 1 μM RA for 0, 0.5, 1 and 2h, then subjected to Western blotting. The fold changes were represented on the top of each band as numerical values that calculated based on the levels of germ cells without culture (0h).

After gonadal sex differentiation, *Stra8* expression occurs only in XX germ cells where it triggers meiotic initiation [6,7,15]. To confirm the sexually dimorphic patterns of *Stra8* transcription in fetal female and male germ cells [17], we first analyzed the levels of *Stra8* transcript in freshly isolated endogenous XX and XY germ cells at E11.5–15.5 (Fig 1A, S2 Table). At E11.5, *Stra8* expression was rarely detected in either XX or XY germ cells. At E12.5, XX germ cells initiated expression of *Stra8* transcripts to a level 3.5x greater than that detected in E11.5 PGCs. At later stages, *Stra8* expression in XX germ cells became further enhanced, reaching a peak at E13.5 (14x higher than that in E11.5) (p < 0.001), which was maintained until E14.5 (p < 0.01 *vs*. E11.5). By E15.5, *Stra8* expression had gradually decreased (p < 0.05). In contrast, in endogenous XY germ cells, on the contrary, *Stra8* transcription was never activated during this same period of E11.5–15.5 (Fig 1A).

### RA selectively stimulates the ERK1/2 pathway in XX germ cells

We next asked whether RA noncanonically stimulates any intracellular signal transduction pathways in germ cells. To answer this question, isolated E12.5 XX germ cells were cultured with 1 μM RA for different time periods (0, 0.5, 1 and 2h), and the phosphorylation levels of three mitogen-activated protein kinases (MAPKs: ERK1/2, JNK and p38 MAPK) and AKT pathways were examined using Western blotting (Fig 1B, S15 Table). After 0.5h of RA treatment, ERK1/2 phosphorylation was slightly increased, and its level was gradually upregulated in a time-dependent manner. After 2h of RA treatment, ERK1/2 phosphorylation was drastically enhanced up to 8.1x compared to that in freshly isolated cells without RA treatment (0h). In contrast, RA treatment did not promote any increase of proteins involved in either the p38 MAPK, JNK or AKT pathways in cultured XX germ cells. These results strongly suggest that RA predominantly stimulates ERK1/2, but not other MAPKs or AKT pathways, in XX germ cells at the time of sex differentiation.

### STRA8 expression is suppressed by inhibiting ERK1/2 activity in XX germ cells

**Fig 2:**
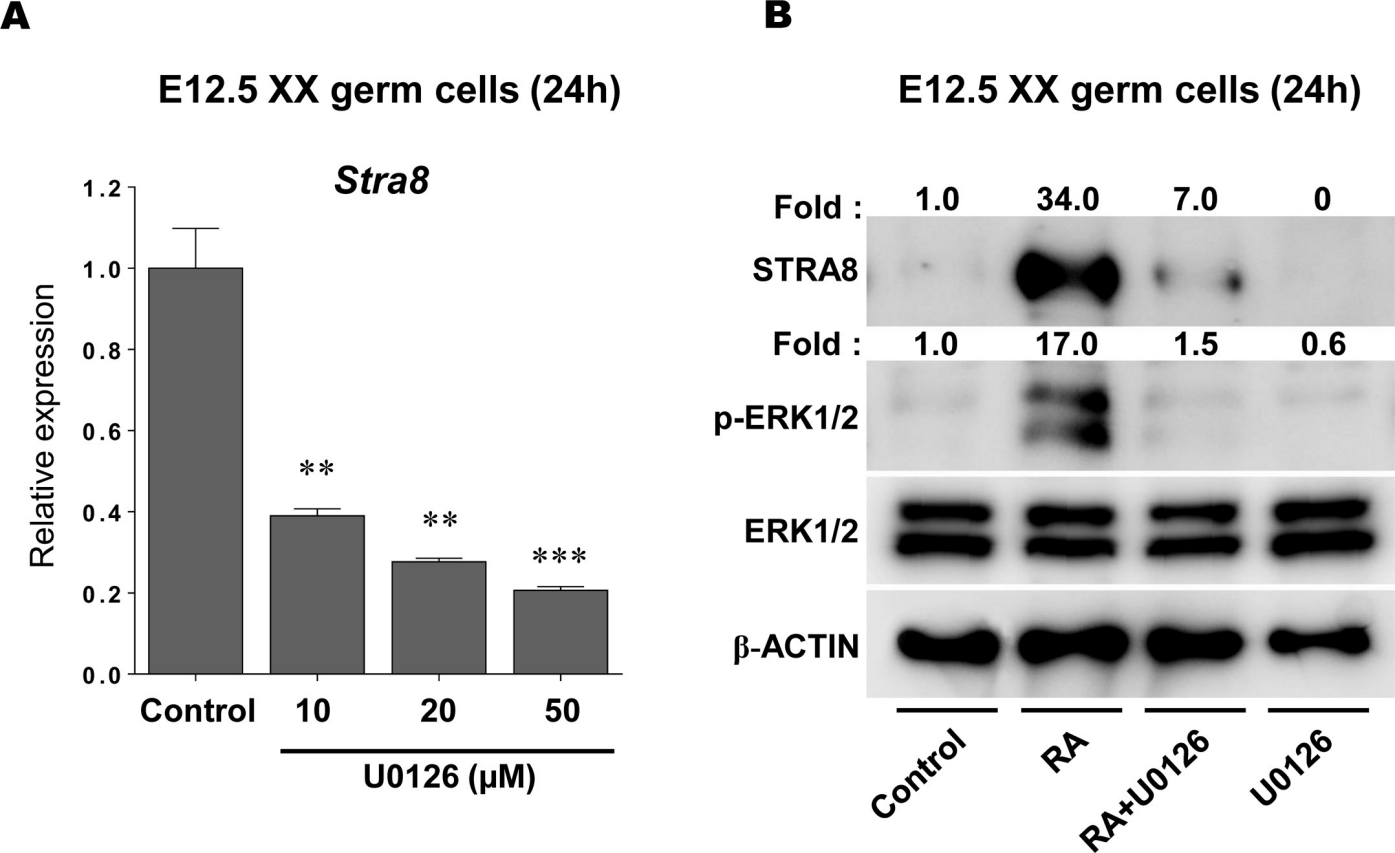
The effect of ERK1/2 signaling activity on *Stra8* expression in XX germ cells. **(**A) To determine the relationship between the ERK1/2 pathway and *Stra8* expression, E12.5 XX germ cells were cultured with the MEK inhibitor (U0126) at different concentrations (0, 10, 20, and 50 μM) for 24h. After culture, samples were subjected to qPCR. Results were normalized to the *β-actin* transcript expression. All expression values were calculated relative to control levels set at 1.0. Data represent the mean ± SEM (n = 3). ** p < 0.01, *** p < 0.001 *vs*. control. (B) To determine the relationship between the RA-stimulated ERK1/2 pathway and STRA8 protein expression, XX germ cells were cultured under the four different conditions (control, 1 μM RA, RA+50 μM U0126, U0126) for 24h. After culture, the cells were subjected to Western blotting to quantify the ERK1/2 phosphorylation and STRA8 protein expression levels. The fold changes of these proteins were represented on the top of each band as numerical values that calculated relative to the control set as 1.0.

To determine whether RA-stimulated ERK1/2 activity contributes to *Stra8* expression in fetal germ cells, we used U0126, a MEK1/2 inhibitor which blocks phosphorylation of ERK1/2 [47]. Isolated XX germ cells were treated with U0126 at different concentrations (0, 10, 20 and 50 μM) for 24h, followed by examination of *Stra8* transcript levels (Fig 2A, S3 Table). In the presence of U0126, *Stra8* transcript levels were effectively suppressed in a dose-dependent manner (p < 0.05–0.01), suggesting an interaction between ERK1/2 phosphorylation and *Stra8* transcription in fetal germ cells. We also confirmed that U0126 at 50 μM effectively suppressed ERK1/2 phosphorylation in XX germ cells (S1 Fig, S15 Table). To further test this hypothesis, isolated E12.5 XX germ cells were cultured with or without 1 μM RA and/or 50 μM U0126 (Fig 2B). These four conditions (RA-/U0126-, RA+/U0126-, RA+/U0126+, RA-/U0126+) were termed control, RA, RA+U0126 and U0126, respectively, and were used for the following experiments. In each case, after 24h of culture, cells were subjected to Western blotting (Fig 2B, S15 Table). In the control condition (RA-/U0126-), both STRA8 expression and ERK1/2 phosphorylation were quite low. In the presence of RA (RA+/U0126-), STRA8 expression was markedly enhanced in cultured XX germ cells (34x higher than the control). At the same time, ERK1/2 activity was obviously upregulated by RA treatment (17x higher than the control). In contrast, RA-stimulated ERK1/2 phosphorylation was strongly suppressed under the RA+U0126 condition (RA+/U0126+) (1.5x higher than the control). Interestingly, RA-stimulated STRA8 expression was also dramatically decreased to 7x higher than the control in the presence of U0126. These data strongly suggest that RA-stimulated ERK1/2 activity directly or indirectly regulates STRA8 protein expression in XX germ cells.

### Transcription levels of *Stra8* and meiotic marker genes in XX germ cells

**Fig 3:**
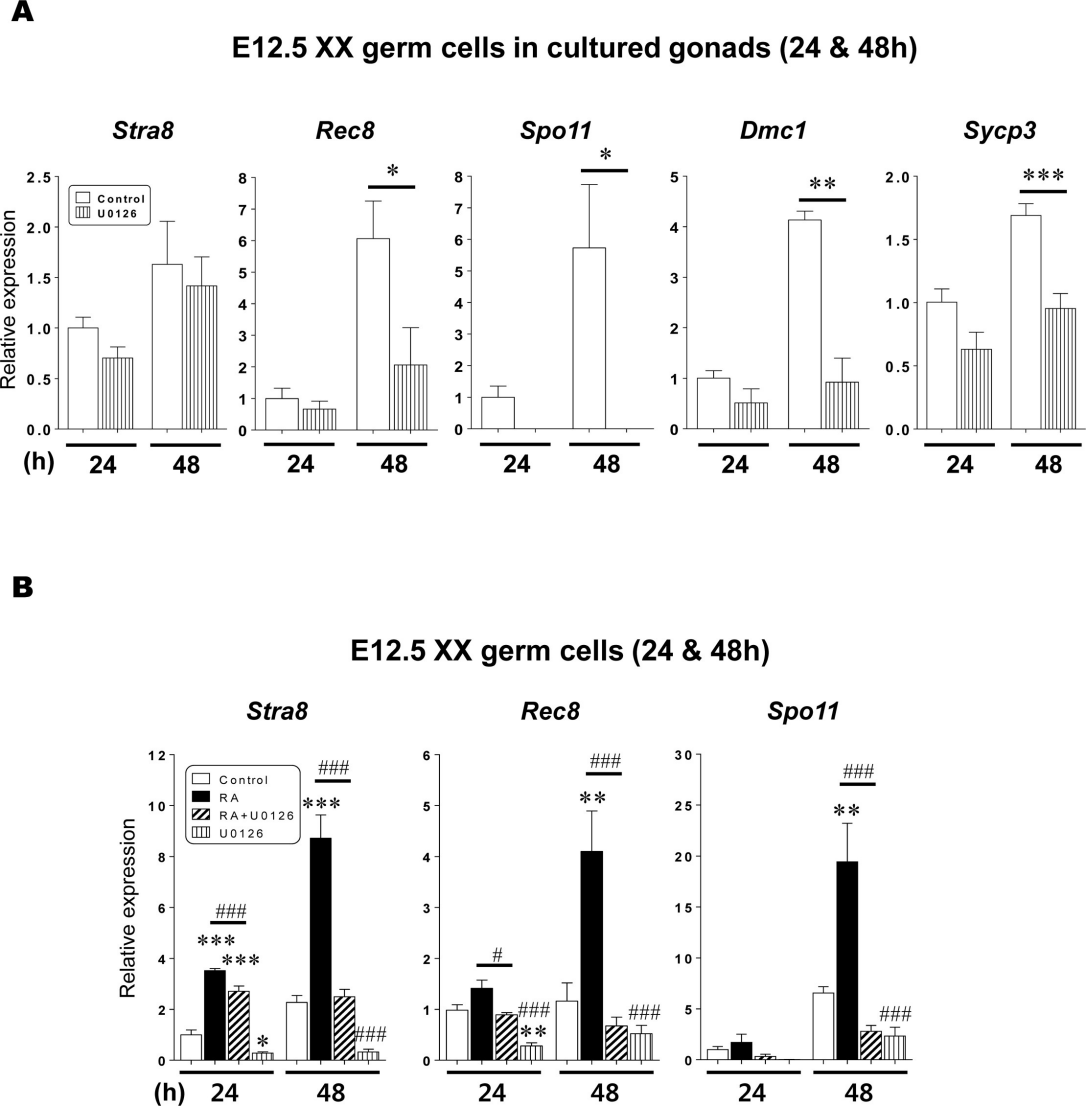
The effect of RA-stimulated ERK1/2 activity on the expressions of *Stra8* and meiotic marker genes in XX germ cells. (A) Female gonads at E12.5 were cultured with 50 μM U0126 (U0126) or without treatment (control) for 24 or 48h. After culture, germ cells were collected to analysis the transcript expressions of *Stra8* and meiotic markers (*Rec8*, *Spo11*, *Dmc1 and Sycp3*). Results were normalized to the *β-actin* transcript expression. All expression values were calculated relative to control levels set at 1.0. Data represent the mean ± SEM (n = 3). * p < 0.05, ** p < 0.01, *** p < 0.001 *vs*. control. (B) Sorted XX germ cells at E12.5 were cultured under four different conditions (control, RA, RA+U0126, U0126) for 24 and 48h. After culture, the cells were subjected to qPCR analysis for *Stra8* and meiotic markers *(Rec8 and Spo11*). Each gene expressions were normalized to the *β-actin* expression. All expression values were calculated relative to control levels set at 1.0. Data represent the mean ± SEM (n = 4). * p < 0.05, ** p < 0.01, *** p < 0.001 *vs*. control; ^#^ p < 0.05, ^###^ p < 0.001 *vs*. RA.

We examined the effect of ERK1/2 activity on meiotic initiation in XX germ cells. E12.5 XX gonads with the mesonephros were cultured with or without 50 μM U0126 for 24 or 48h. After culture, the gonads were dissociated into singe-cell suspensions to collect GFP-positive germ cells. These cells were subjected to the qPCR analysis for *Stra8* and four meiotic marker genes (*Rec8*, *Spo11*, *Dmc1*, and *Sycp3*) (Fig 3A, S4 Table). In the control germ cells, *Stra8* expression after 48h of culture was upregulated to 1.6x higher than that at 24h culture. At the same time, the transcript levels of all meiotic marker genes were entirely promoted (1.7–6.0x) after 48h of culture compared to those after 24h of culture. In the presence of U0126, *Stra8* transcript levels after 24 or 48h of culture were slightly but consistently decreased compared with those in the controls, respectively (p > 0.05). Importantly, U0126 treatment in the XX gonads also suppressed the transcript levels of the four meiotic markers after 24h of culture (Fig 3A). In particular after 48h of culture, all meiotic marker expressions were significantly suppressed compared to the controls in the gonadal germ cells (p < 0.05–0.001). To test the influence of U0126 on the cell viability, we also analyzed the expression of *mouse Vasa homologue* (*Mvh*) transcripts as a control germ cell marker [48]. *Mvh* expression levels were very similar between the control and U0126 conditions both at 24 (relative expression average 1.00 vs. 1.12) or 48h (1.63 vs. 1.38), and found no statistical differences (p > 0.05) (S2A Fig, S12 Table). Taken together, these results suggested that ERK1/2 activity plays a role in entry of XX germ cells into meiosis. To further test a direct effect of U0126 on the germ cells, we next completely neglected the influence of the mesonephros and gonadal somatic cells. Isolated E12.5 XX germ cells were cultured under four conditions (control, RA, RA+U0126, and U0126). After 24 or 48h of culture, morphologically healthy cells (200–300 cells per set) were collected to analyze the transcript levels of *Stra8* and two meiotic marker genes (*Rec8 and Spo11*) (Fig 3B, S5 Table). In XX germ cells after 24h of culture, U0126 treatment significantly diminished *Stra8* expression compared to the control (p < 0.05). In contrast, RA treatment significantly upregulated *Stra8* transcript level to 3.5x higher than the control (p < 0.001). Under the RA+U0126 condition, RA-stimulated *Stra8* expression was significantly suppressed (to approximately 25% of that under the RA condition) (p < 0.001). After 48h of culture, U0126 significantly suppressed *Stra8* expression compared to the control condition (p < 0.05). In the presence of RA, *Stra8* expression level was further increased to 8x higher than the control at 24h (p < 0.001). However, U0126 treatment significantly suppressed RA-induced *Stra8* expression to the control level at 48h (p < 0.001). Meiotic marker gene expressions were also dramatically affected by U0126 treatment (Fig 3B). After 24h of culture, both *Rec8* and *Spo11* expression levels were slightly increased in the presence of RA (p > 0.05), but these expressions were suppressed by U0126 treatment (*Rec8*, p < 0.05; *Spo11*, p > 0.05). After 48h of culture, RA treatment significantly enhanced *Rec8* and *Spo11* expression levels (4.1x and 19.4x higher than the controls, respectively) (p < 0.01). Importantly, U0126 treatment significantly decreased RA-induced meiotic marker expressions to lower than their controls (p < 0.001). Under the U0126 condition, both gene expression levels were also diminished compared with those in the controls (p > 0.05). In XX germ cells at 24h, the *Mvh* expression levels under the four culture conditions were very similar (1.00, 0.90, 0.93, 0.77, respectively) regardless with or without U0126 treatment (p > 0.05) (S2B Fig, S13 Table). At 48h, the average expression levels in the control (2.06) and RA (1.92) conditions were higher compared to those in RA+U0126 (0.84) and U0126 (0.70) conditions. Importantly, there were no significant differences among four conditions at 48h (p > 0.05), and U0126 treated groups (RA+U0126, U0126) at 48h have maintained the similar expression levels as those at 24h (S2B Fig, S13 Table). In conclusion, these results strongly suggest that RA-stimulated ERK1/2 activity directly regulates transcription of the *Stra8* and meiotic marker genes in XX germ cells.

### Meiotic initiation is suppressed by inhibiting ERK1/2 activity in XX germ cells

**Fig 4:**
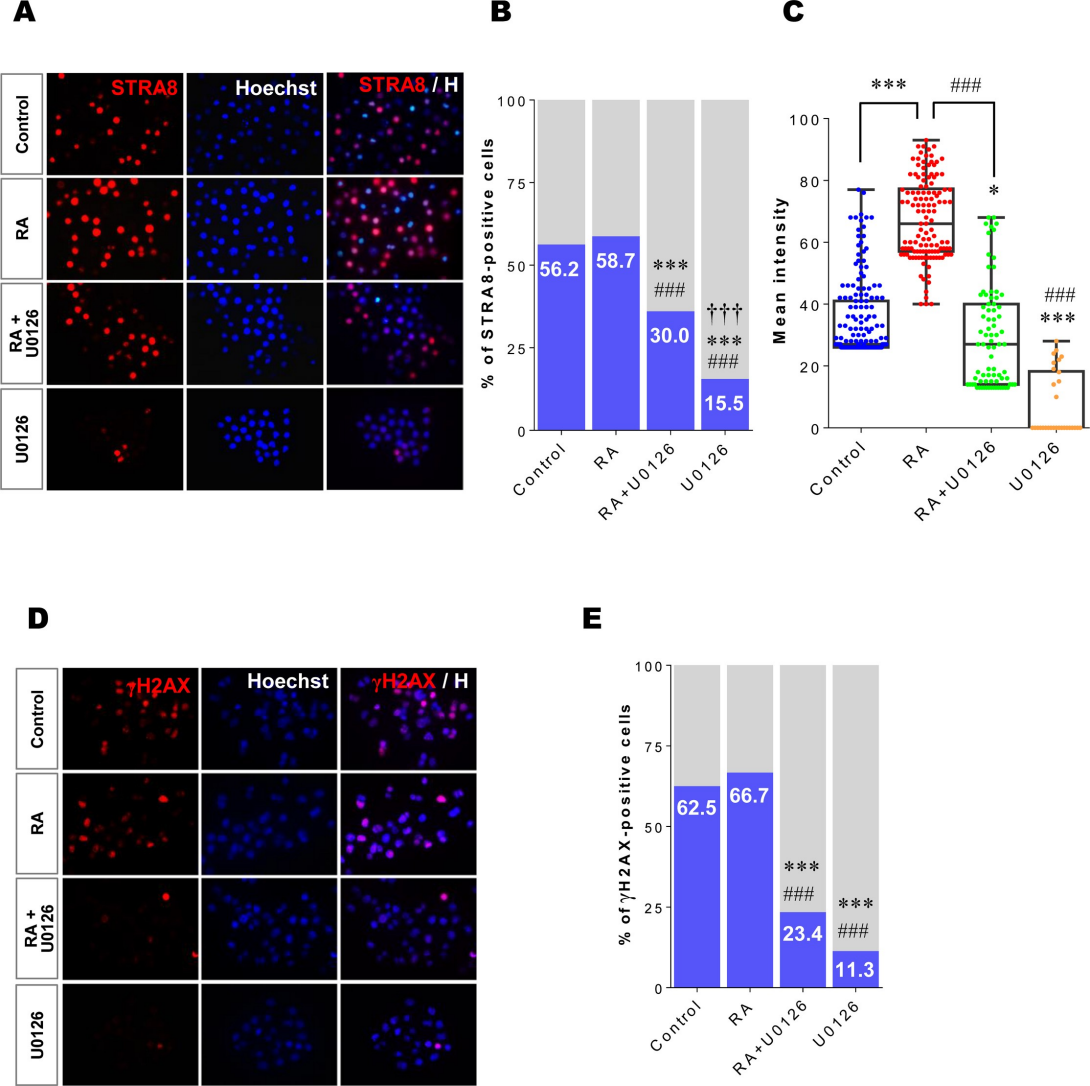
Inhibiting ERK1/2 activity suppresses STRA8 protein expression and meiotic initiation in XX germ cells. Isolated XX germ cells at E12.5 were cultured under four different conditions (control, RA, RA+U0126, U0126) for 24h. (A) After culture, germ cells were immunostained with an anti-STRA8 antibody (red) and counterstained with Hoechst33342 (blue). (B) The ratio (%) of STRA8-positive cells in each culture condition was estimated using ImageJ software. *** p < 0.001 *vs*. control; ^###^ p < 0.001 *vs*. RA; ^†††^ p < 0.001 *vs*. RA+U0126. (C) The fluorescent intensity of STRA8 protein expression in each positive cell was quantified using ImageJ software. The results were represented as a box-whisker plot. Each dot represents a single analyzed germ cell. Each box indicates the middle 50% of the data and midline in the box indicates the median. The whiskers extend to the most extreme data point, which is no more than 1.5 times the quartile range. * p < 0.05, *** p < 0.001 *vs*. control; ^###^ p < 0.001 *vs*. RA. (D) To examine the entry into meiosis, cultured XX germ cells were stained with an antibody against γH2AX (red) and counterstained with Hoechst33342 (blue). (E) The ratio (%) of γH2AX-positive cells in each condition was estimated using ImageJ software. *** p < 0.001 *vs*. control; ^###^ p < 0.001 *vs*. RA.

To confirm the effect of the RA-stimulated ERK1/2 pathway on STRA8 protein expression, E12.5 XX germ cells were cultured under the four culture conditions for 24h. After culture, XX germ cells (200–300 cells per sample) were immunostained with antibody against STRA8 (Fig 4A). First, we examined the proportion of STRA8-positive cells under the different culture conditions (Fig 4B, S6 Table). In the control, more than half of the cultured germ cells (56.2%) were STRA8-positive. In the presence of RA, the ratio of STRA8-positive cells was similar to the control (58.7%) (p > 0.05). These results suggest that RA treatment does not promote STRA8-negative XX germ cells to become STRA8-positive at E12.5. In contrast, the proportion of STRA8-positive cells (30.0%) was significantly reduced under the RA+U0126 condition compared to that under either the control or RA- treated conditions (p < 0.001). Under the U0126 condition, only 15.5% of the examined XX germ cells were STRA8-positive (p < 0.001). These results indicate that the ERK1/2 pathway contributes to regulation of STRA8 protein expression in cultured XX germ cells. Although RA treatment did not increase the proportion of STRA8-positive XX germ cells (Fig 4B), the fluorescence levels in the positive cells cultured under the RA condition were much stronger compared with those in cells cultured under the other three conditions (Fig 4A). Therefore, we next measured the fluorescence intensity of STRA8 protein expression in positive-cells individually using ImageJ software (National Institutes of Health, Bethesda, MD, USA) (Fig 4C, S7 Table). For this purpose, we evaluated fluorescence intensity in STRA8 positive cells as follows: control, 171 cells; RA, 126 cells; RA+U0126, 80 cells; and U0126, 30 cells. Without RA treatment (control), the mean value of STRA8 fluorescence intensity was 34.63 ± 0.97 (median is inside line of box 27.0). In the presence of RA, the STRA8 fluorescence intensity was significantly enhanced to 67.34 ± 1.17 (median 66.0) in XX germ cells, almost twice that of the control cells (p < 0.001). When ERK1/2 activity was inhibited by U0126 (RA+U0126), RA-stimulated expression of STRA8 was significantly suppressed to 29.43 ± 1.86 (median 27.0; p < 0.001), a level that was significantly lower than that in the control (p < 0.05). Furthermore, the cells in the U0126 condition showed severely diminished STRA8 fluorescence intensity (mean 7.30 ± 1.87; median 0.00) compared with either the control (p < 0.001) or RA-only (p < 0.001) conditions. Taken together, these results suggest that RA-enhanced STRA8 protein levels are controlled by the RA-stimulated EK1/2 pathway.

We next determined the relationship between the ERK1/2 pathway and entry into meiosis. XX germ cells at E12.5 were cultured under the same four different conditions and samples of about 100 cells per group were then placed on glass slides to be subjected to immunostaining with an antibody against γH2AX protein, a biomarker of the meiotic DNA double-strand breaks that occur at the leptotene stage of prophase I in meiosis (Fig 4D) [49]. Similar to the results shown in Fig 4B, the population of γH2AX-positive cells in the control (62.5%) group was not increased even in the presence of RA (66.7%) (Fig 4E, S8 Table), which suggests that RA treatment does not promote entry of XX germ cells into meiosis at E12.5. Interestingly, a previous study also determined that RA treatment does not accelerate the timing of meiotic entry in isolated E12.5 germ cells [18]. In contrast, inhibition of ERK1/2 phosphorylation by U0126 significantly reduced the proportion of γH2AX-positive germ cells in both the RA+U0126 (23.4%) and U0126 (11.3%) conditions compared with those in the control (p < 0.001) and RA conditions (p < 0.001). Thus, STRA8- and meiotic-positive cell numbers were well correlated under all four conditions (Fig 4B and 4E). Taken together, our results support the conclusion that the RA-mediated ERK1/2 pathway regulates STRA8 protein expression, which, in turn, induces entry of XX germ cells into meiosis.

### RA-induced meiotic initiation is suppressed by inhibiting ERK1/2 activity in XY germ cells

**Fig 5:**
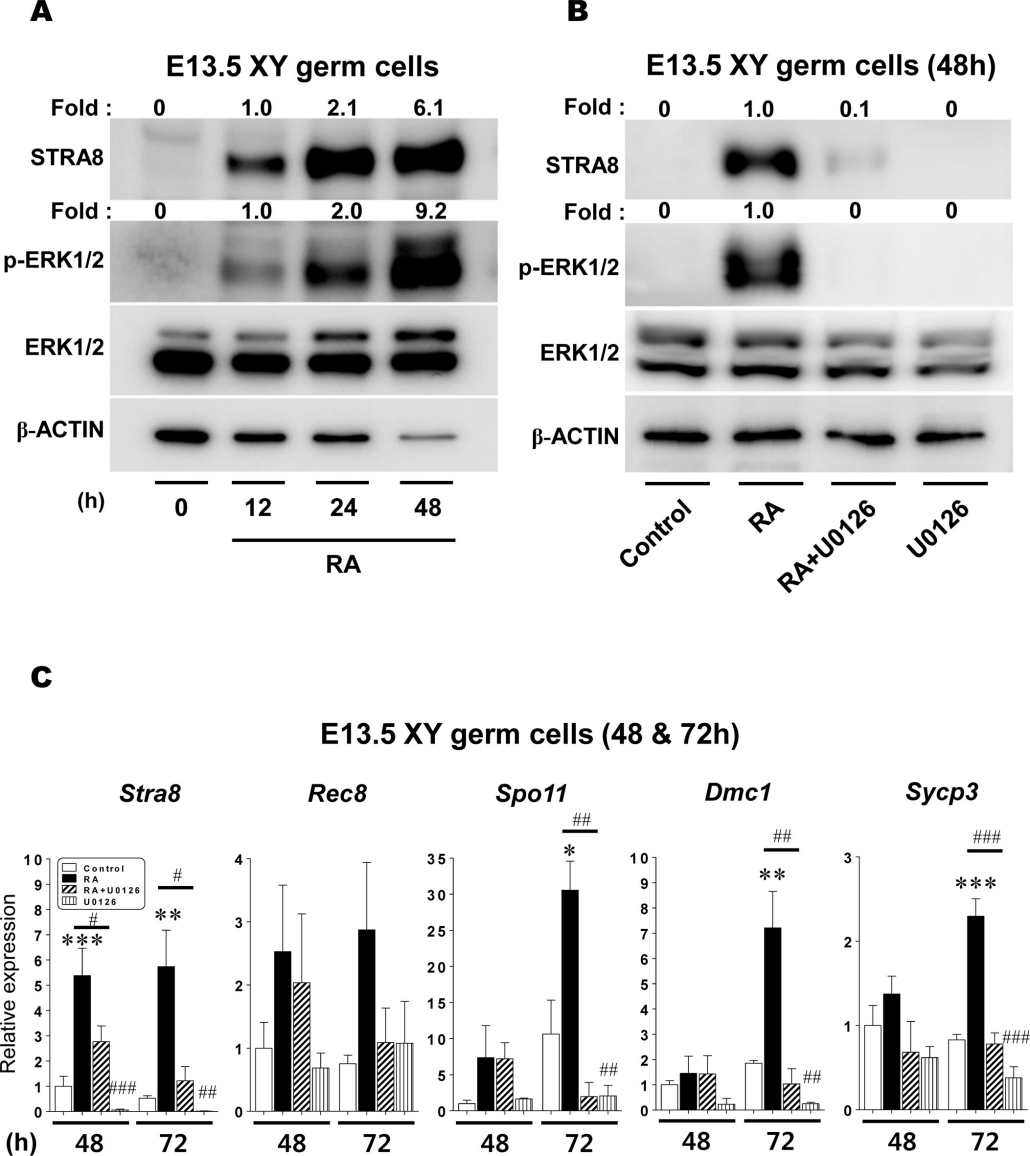
The effect of RA-stimulated ERK1/2 activity on the meiotic initiation in XY germ cells. (A) Isolated XY germ cells at E13.5 were cultured with 1μM RA for 0, 12, 24, and 48h, and the ERK1/2 phosphorylation and STRA8 protein expression were then analyzed using Western blotting. (B) Isolated XY germ cells at E13.5 were cultured under four different conditions (control, RA, RA+U0126, U0126). After 48h culture, the cells were subjected to Western blotting to quantify the ERK1/2 phosphorylation and STRA8 protein expression levels. The fold changes of these proteins were represented on the top of each as numerical values that calculated relative to either 0h level (A) or the control (B) set as 1.0. (C) Isolated E13.5 XY germ cells were cultured under four different conditions (control, RA, RA+U0126, U0126) for 48 and 72h. After culture, the cells were subjected to qPCR analysis to determine the transcript levels of *Stra8* and meiotic marker genes (*Rec8*, *Spo11*, *Dmc1 and Sycp3*). The expression levels were normalized to *β-actin* mRNA expression. All expression values were calculated relative to control levels set at 1.0. Data represent the mean ± SEM (n = 3–6). * p < 0.05, ** p < 0.01, *** p < 0.001 *vs*. control; ^#^ p < 0.05, ^##^ p < 0.01, ^###^ p < 0.001 *vs*. RA.

We have previously reported that XY germ cells isolated at E13.5 autonomously progress through the male pathway in culture without somatic support [46]. Our previous study also determined that RA treatment directly induces E13.5 XY germ cells to express *Stra8* and initiate meiosis [13]. In the present study, we examined whether or not RA-induced STRA8 expression in XY germ cells is regulated by the RA-stimulated ERK1/2 pathway. To determine the relationship between STRA8 expression and ERK1/2 activity, E13.5 XY germ cells were cultured with 1 μM RA for 0, 12, 24 and 48h prior to Western blotting (Fig 5A). As expected, RA treatment gradually induced STRA8 expression in XY germ cells in a time-dependent manner. Interestingly, RA treatment also gradually promoted ERK1/2 activity in proportion to RA-induced STRA8 levels in cultured XY germ cells (Fig 5A, S15 Table).

Next, we examined the direct interaction between RA-stimulated ERK1/2 activity and STRA8 expression in XY germ cells. XY germ cells at E13.5 were cultured for 48h under the same four basic conditions shown in Fig 2B (Fig 5B, S15 Table). In the control condition, neither STRA8 expression nor ERK1/2 phosphorylation was observed in XY germ cells. In contrast, RA treatment dramatically activated STRA8 protein expression as well as ERK1/2 phosphorylation in XY germ cells. In the presence of U0126 (RA+U0126), RA-stimulated ERK1/2 phosphorylation was effectively suppressed. At the same time, U0126 treatment significantly decreased the RA-induced STRA8 expression to only 10% of the level observed in the RA condition. These results suggest that the RA-stimulated ERK1/2 pathway also controls the RA-induced STRA8 expression in cultured E13.5 XY germ cells.

We next examined *Stra8* and four meiotic marker (*Rec8*, *Spo11*, *Dmc1*, and *Sycp3*) transcript levels in E13.5 XY germ cells cultured for 48 or 72h under the four different conditions (Fig 5C, S9 Table). After 48h of culture, RA treatment significantly upregulated *Stra8* transcription to 5.4x higher than the control (p < 0.001). However, U0126 significantly suppressed RA-induced *Stra8* transcript levels (to approximately 50% of that under the RA condition) (p < 0.05). U0126 treatment suppressed *Stra8* expression relative compared to the control germ cells (p > 0.05). After 72h of culture, RA treatment maintained a similar *Stra8* transcript level to 48h. RA-induced *Stra8* expression was also significantly diminished by the U0126 treatment (RA+U0126). In contrast, the expression levels of meiotic marker genes at 48h were only slightly elevated in the presence of RA, and there was no significant difference compared to their controls, respectively (Fig 5C). After 72h of culture, RA treatment significantly enhanced the expression levels of *Spo11*, *Dmc1* and *Sycp3*,compared to their controls (30.6x, 7.2x and 2.3x, respectively) (p < 0.05–0.001). Importantly, U0126 treatment dramatically suppressed all marker gene expressions induced by RA compared to the controls (p < 0.01–0.001). On the contrary, *Mvh* expression under the four culture conditions was very similar at 48h (p > 0.05) (S3 Fig, S14 Table). At 72h, the average expression levels were a little more varied (1.18, 1.61, 0.85, and 0.68, respectively). Although one significant difference was detected between RA and U0126 conditions (p < 0.05), there were no any other statistical differences between each condition in cultured XY germ cells (p > 0.05) (S3 Fig, S14 Table). These data strongly support that RA-stimulated ERK1/2 activity plays a role in regulating the transcription of *Stra8* and meiotic marker genes in XY germ cells.

### The RA-stimulated ERK1/2 pathway regulates STRA8 expression and meiotic initiation in XY germ cells

**Fig 6:**
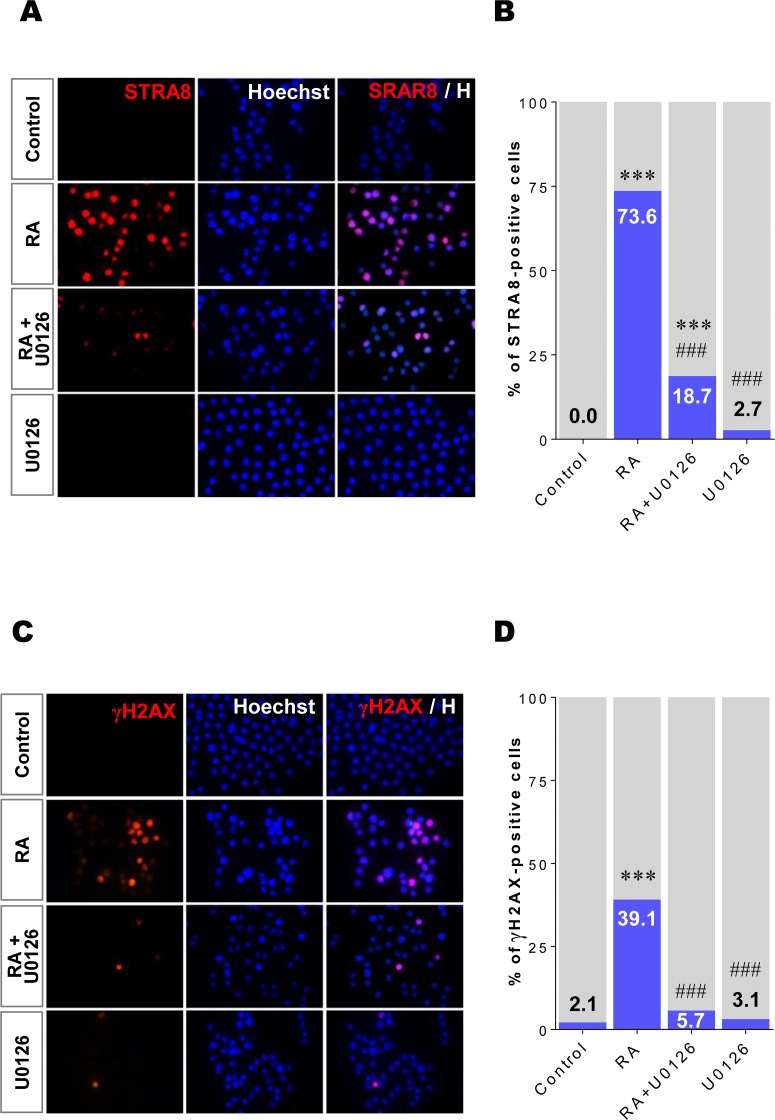
Inhibiting ERK1/2 activity suppresses RA-induced STRA8 protein expression and meiotic initiation in XY germ cells. Isolated XY germ cells at E13.5 were cultured under four conditions (control, RA, RA+U0126, U0126) for 48h. (A) The cultured cells were immunostained with the anti-STRA8 antibody and counterstained with Hoechst33342 (blue). (B) The ratio (%) of STRA8-positive cells was estimated in each condition. *** p < 0.001 *vs*. control; ^###^ p < 0.001 *vs*. RA. (C) The cultured germ cells were immunostained with the anti-γH2AX antibody (red) and counterstained with Hoechst33342 (blue). (D) The ratio (%) of γH2AX-positive cells was estimated in each condition. *** p < 0.001 *vs*. control; ^###^ p < 0.001 *vs*. RA.

We further tested whether the RA-stimulated ERK1/2 pathway regulates STRA8 protein expression and entry into meiosis in E13.5 XY germ cells (Fig 6). Sorted XY germ cells were cultured for 48h under the same basic four conditions. Samples of about 300–400 XY germ cells were collected and placed on glass slides to be subjected to immunostaining with antibody against STRA8 protein (Fig 6A) to obtain the numbers of STRA8-positive cells (Fig 6B, S10 Table). Without RA treatment (control), XY germ cells never became STRA8-positive in culture (0%) (Fig 6A and 6B). In the presence of RA, 73.6% of the tested XY germ cells strongly expressed STRA8 (p < 0.001). However, U0126 treatment significantly suppressed RA-induced STRA8 expression and only 18.7% of analyzed XY germ cells were STRA8-positive (p < 0.001) (Fig 6A and 6B). Further, STRA8-positive cells were rarely observed in the presence of U0126 only (2.7%, p > 0.05 *vs*. control). These data confirm that the RA-stimulated ERK1/2 pathway regulates RA-stimulated STRA8 expression in E13.5 XY germ cells in culture.

To determine if the RA-stimulated ERK1/2 pathway impacts meiotic initiation in XY germ cells, E13.5 XY germ cells were cultured under the same four conditions. After culture, samples of 200–300 XY germ cells were collected and immunostained with γH2AX antibody (Fig 6C) to determine the number of cells that had entered meiosis (Fig 6D, S11 Table). Interestingly, the expression patterns of γH2AX appeared to correlate with those of STRA8 in cultured XY germ cells (Fig 6B and 6D). Without RA, γH2AX-positive cells were rarely detected in the control and U0126 treatment conditions (2.1% and 3.1%, respectively). In the presence of RA, about 40% of the germ cells entered meiosis (p < 0.001). Under U0126 treatment, most of the RA-induced meiotic cells suppressed γH2AX expression, and only 5.7% entered meiosis (p < 0.001). Taken together, our results clearly demonstrate that the RA-stimulated ERK1/2 pathway plays a critical role in regulating STRA8 expression and entry into meiosis in cultured E13.5 XY germ cells.

## Discussion

Recent studies in somatic cells have proposed that RA noncanonically activates intracellular signal transduction pathways to regulate transcription of RA target genes [37,50,51]. In this study, we examined 1) whether RA noncanonically activates any MAPK pathways in fetal germ cells, and 2) if this is the case, whether the RA-stimulated signaling pathway regulates *Stra8* expression and entry into meiosis in fetal germ cells. We found that RA treatment selectively stimulates the ERK1/2 pathway in XX germ cells at E12.5. We further demonstrated that RA-induced *Stra8* expression (at both the mRNA and protein levels) and several meiotic marker expressions were significantly suppressed by inhibiting the RA-stimulated ERK1/2 pathway in cultured germ cells, regardless of their sexual phenotype. Our findings suggest a new concept that *Stra8* expression may be controlled not only by the canonical activity of RA via nuclear RARs, but also by the noncanonical activity of RA via the ERK1/2 pathway in fetal germ cells.

Our initial interest was whether the noncanonical route of RA activity originally evidenced by somatic cell studies is also utilized in fetal germ cells for meiotic initiation or not. Interestingly, treatment with RA predominantly enhanced the ERK1/2 pathway, but not the p38 MAPK, JNK or AKT pathways in E12.5 XX germ cells in culture. Recent studies in the somatic cells have revealed a novel biological activity of RA via signal transduction pathways, suggesting that RA induces the selective activation of multiple signal transduction pathways [28,29]. In particular, the RA-induced ERK1/2 activation has been most widely reported in various cell types [21,22,32,35,39,52–55], including postnatal mouse spermatogonia [40]. In embryonic stem (ES) cell-derived embryoid bodies (EBs), RA treatment induces differentiation into various cell lineages including adipogenesis, neurogenesis and myogenesis. Bost et al. (2002) reported that the ERK inhibitor treatment suppressed adipocyte differentiation, but did not affect other lineage differentiation in the EBs [39]. This study suggests that the RA-stimulated ERK pathway selectively induces adipocyte differentiation in ES cells [39]. In the nervous system, RA is known as a potent regulator to induce neurite outgrowth and neuronal differentiation. In a rat derived A126-1B2 cells, e.g., RA treatment activates *c-fos* gene expressions to induce neuronal differentiation [56]. A previous study suggested that the RA-stimulated ERK1/2 pathway controls these gene expressions to induce neural differentiation [32]. In A126-1B2 cells, RA treatment activated both the ERK1/2 signaling and cAMP-response element-binding protein (CREB), a transcription factor which regulates the transcription of genes including *c-fos* and *c-jun* [32]. It was suggested that the RA-stimulated ERK1/2 pathway controls neuronal differentiation through CREB activation because U0126 treatment abolished RA-induced CREB phosphorylation [32]. In the human leukemia HL-60 cell line, RA-stimulated ERK2 signaling directs myeloid differentiation [55]. Importantly, RA selectively stimulates ERK but not other MAPK pathways (JNK/SAPK or p38) for myeloid differentiation [22]. Our present study also demonstrated that RA selectively stimulates the ERK1/2, but not other MAPK or AKT pathways in fetal germ cells. Taken together, these previous studies and our result strongly suggest that RA noncanonically stimulates the ERK1/2 pathways to regulate a large spectrum of cellular activities and functions, including entry of germ cells into meiosis.

We found that the ERK phosphorylation inhibitor (U0126) effectively suppressed RA-induced *Stra8* expression (both at the mRNA and protein levels) in E12.5 XX germ cells and E13.5 XY germ cells in culture. Thus, our results are consistent with the novel idea that the RA-stimulated ERK1/2 pathway contributes to *Stra8* expression in fetal germ cells. Using postnatal mouse spermatogonia, Pellegrini et al. (2008) determined that both RA and Kit Ligand (KL) increased the mRNA expression of *Stra8* and *Dmc1* [40]. By inhibiting Kit signaling activity, the meiotic cell number was decreased, suggesting Kit dependent downstream pathways (MARK, PI3K) contribute to meiotic initiation. Actually, either MEK inhibitor (U0126) or PI3K inhibitor (LY294002) treatment for 48h suppressed RA-induced meiosis in the postnatal male germ cells [40]. Although they did not analyze the direct relationship between MAPK and *Stra8* expression, their study strongly supports the idea that noncanonical activity of RA controls meiotic initiation. Very recently, it was reported that inactivation of *Wdr62*, which encodes a scaffold protein in the JNK signaling pathway, caused meiotic initiation defects in mice [41]. The fetal gonads treated with a JNK inhibitor suppressed *Stra8* and meiotic marker expressions, suggesting *Wdr62* is involved in RA-induced meiotic initiation via activating JNK pathway [41]. This is another example that the intracellular signaling pathways contribute to meiotic initiation in the germ cells. Previous evidence has shown that the RA bound RAR/RXR heterodimer canonically binds to RAREs to regulate RA target gene expression [23,57]. As a typical RA target gene, murine *Stra8* contains two putative RAREs (DR2 and IR5) within a 400 bp promoter region upstream from the transcription start site [26]. In the prepubertal mouse testis, a luciferase reporter assay showed that the 400 bp promoter region is sufficient to direct *Stra8* transcriptional expression [52]. Using a ChIP assay, Raverdeau et al. (2012) have detected direct binding of all RAR isotypes to the RARE (DR2) in the *Stra8* promoter region in the postnatal testes at 8 dpp [9]. These results suggest that RA-induced *Stra8* expression is canonically mediated by nuclear RARs in germ cells. In the fetal ovary at E13.5, in contrast, a similar ChIP study reported that the binding affinity of RARs (α, β, γ) to RAREs in the *Stra8* promoter is either very low or not detectable [27]. However, RAR antagonists successfully suppressed the transcriptional activity of *Stra*8 and meiotic initiation in isolated germ cells and gonadal germ cells *in vitro* [6,7,11,43]. Based on these studies, it seems that the biological activity of RA is mediated by nuclear RARs in fetal germ cells. Importantly, our new findings suggest an alternate route of RA signaling mediated by the ERK pathway to control *Stra8* expression. Actually, previous studies in somatic cells have suggested that expression of RA target genes is regulated by RA-stimulated signal transduction cascades. Thus, in both mouse embryonic fibroblasts and human breast cancer cells, RA treatment activates the p38 MAPK pathway to enhance transcription of *Cyp26A1*, one of the RA target genes [37]. In embryonic fibroblasts, the RA-stimulated p38 MAPK pathway regulates transcription of another RA target gene, *RARβ2*, which may be involved in epigenetic events, such as chromatin modification and remodeling [37]. The molecular mechanism of the RA-stimulated signal transduction cascades in regulating transcription of the target genes remains unclear. In general, phosphorylated ERK1/2 is translocated into the nucleus to stimulate multiple downstream target molecules [21,32,34,35,58–60]. Therefore, one idea is that RA-stimulated kinase signaling might be crucial for the fine tuning of the appropriate physiological activity of their target genes. For example, activated ERK1/2 signaling phosphorylates mitogen- and stress-activated protein kinase 1 (MSK1), which can phosphorylate several nuclear proteins [58–60]. Specifically, activated MSK1 phosphorylates histone H3 at Ser28 residues, which eventually dissociates the polycomb repressive complexes (PRCs) to predispose a transcriptionally active state [51,61]. PRCs are involved in gene silencing through histone posttranslational modifications [62,63]. Therefore, it appears that the RA-stimulated ERK1/2 pathway may play an important role in epigenetic regulation of gene expression. Recently Yokobayashi et al. (2013) revealed that PRC1, one of the PRCs, controls the timing of *Stra8* transcriptional activation in female gonads [64]. In PRC1-deficient germ cells, *Stra8* transcription was prematurely activated at E11.5, and meiotic-related genes were expressed earlier than usual. These authors speculated that PRC1 maintains repression of *Stra8* and early meiosis programs until RA signaling has reached a certain threshold in female gonads [64]. In conclusion, *Stra8* expression appears to be epigenetically regulated via histone modifications and chromatin accessibility, which may be associated with the function of RA-induced signal transduction cascades.

**Fig 7:**
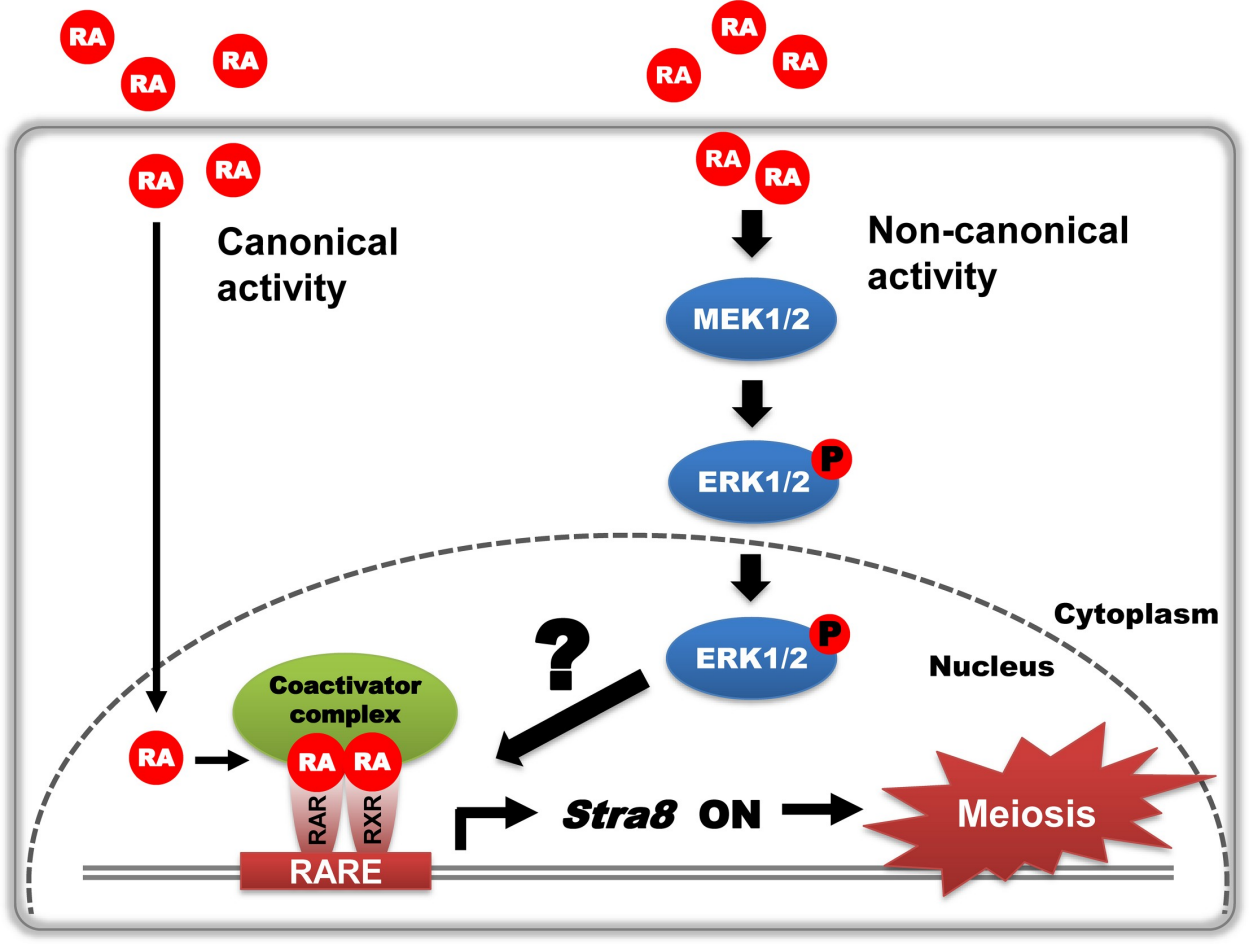
RA noncanonically activates the ERK1/2 pathway to regulate *Stra8* expression and meiotic initiation in fetal germ cells. In general, RA canonically binds to nuclear RARs to control the transcriptional expression of RA target genes. Based on our results, we propose a novel concept that RA signaling also functions via an alternate signal transduction cascade in the cytoplasm, independent of the canonical pathway mediated by nuclear RARs, to control meiosis. In isolated XX and XY germ cells, external RA noncanonically stimulates the ERK1/2 signal transduction pathway in the cytoplasm. The phosphorylated ERK1/2 moves to the nucleus and contributes to *Stra8* expression to stimulate meiotic initiation. Although the details of the molecular features of this proposed alternate RA-stimulated ERK1/2 pathway remain unclear, it is possible that crosstalk between the canonical and noncanonical activities of RA impact the transcriptional regulation of *Stra8* to induce entry into meiosis.

Using our germ cell culture system, we identified that RA stimulates the ERK1/2 activity via a noncanonical pathway in both XX and XY germ cells. Furthermore, we demonstrated that the RA-stimulated ERK1/2 pathway contributes to *Stra8* expression at both the mRNA and protein levels, resulting in regulating entry into meiosis. Thus, we propose the novel concept that RA signaling functions via an alternate signal transduction cascade to control *Stra8* expression and meiosis, independent of the canonical pathway mediated by nuclear RARs (Fig 7). Although details of the molecular features of this proposed alternate RA-stimulated ERK1/2 pathway remain unclear, it is possible that crosstalk between the canonical and noncanonical activities of RA impact the transcriptional regulation of *Stra8* to induce entry into meiosis.

## Supporting information

S1 Table Antibodies information used for Western blotting and immunochemical staining. (PDF)

S2 Table The raw data for qPCR of Fig 1A. (PDF)

S3 Table The raw data for qPCR of Fig 2A. (PDF)

S4 Table The raw data for qPCR of Fig 3A. (PDF)

S5 Table The raw data for qPCR of Fig 3B. (PDF)

S6 Table The raw data for qPCR of Fig 4B. (PDF)

S7 Table The raw data for qPCR of Fig 4C. (PDF)

S8 Table The raw data for qPCR of Fig 4E. (PDF)

S9 Table The raw data for qPCR of Fig 5C. (PDF)

S10 Table The raw data for qPCR of Fig 6B. (PDF)

S11 Table The raw data for qPCR of Fig 6D. (PDF)

S12 Table The raw data for qPCR of S2A Fig. (PDF)

S13 Table The raw data for qPCR of S2B Fig. (PDF)

S14 Table The raw data for qPCR of S3 Fig. (PDF)

S15 Table O.D. values for Western blot (Figs 1B, 2B, 5A and 5B and S1 Fig). For signal transduction pathways, the *β*-ACTIN-normalized phospho/total protein ratio was set as the O.D. value of each phosphorylated protein. The O.D. value of STRA8 was determined by normalizing to the *β*-ACTIN. The data represent the mean ± SD of two independent WB experiments.
(PDF)

S1 Fig The effect of U0126 on the ERK1/2 phosphorylation in XX germ cells. To evaluate the effect of U0126 on the ERK1/2 phosphorylation, XX germ cells at E12.5 were cultured with a MEK inhibitor (U0126) at different concentrations (0, 10, 20, and 50 μM) for 1h. After culture, germ cells were subjected to Western blotting to quantify the ERK1/2 phosphorylation. The levels of protein bands were quantified by densitometry and represented on the top of each band as numerical values that calculated relative to the control set as 1.0.
(PDF)

S2 Fig The effect of RA-stimulated ERK1/2 activity on the expressions of *Mvh* in XX germ cells. (A) Female gonads at E12.5 were cultured with 50 μM U0126 (U0126) or without treatment (control) for 24 or 48h. After culture, germ cells were collected to analysis the transcript expressions of *mouse Vasa homolog* (*Mvh*). Results were normalized to the *β -actin* transcript expression. All expression values were calculated relative to control levels set at 1.0. Data represent the mean ± SEM (n = 3). (B) Sorted XX germ cells at E12.5 were cultured under four different conditions (control, RA, RA+U0126, U0126) for 24 and 48h. After culture, the cells were subjected to qPCR analysis for *Mvh*. Each gene expressions were normalized to the *β-actin* expression. All expression values were calculated relative to control levels set at 1.0. Data represent the mean ± SEM (n = 4).
(PDF)

S3 Fig The effect of RA-stimulated ERK1/2 activity on the expressions of *Mvh* in XY germ cells. Isolated E13.5 XY germ cells were cultured under four different conditions (control, RA, RA+U0126, U0126) for 48 and 72h. After culture, the cells were subjected to qPCR analysis to determine the transcript levels of *Mvh*. The expression levels were normalized to *β -actin* mRNA expression. All expression values were calculated relative to control levels set at 1.0. Data represent the mean ± SEM (n = 3–4). ^#^ p < 0.05 *vs*. RA.
(PDF)
