# Supplementary material for: Retinoic acid-stimulated ERK1/2 pathway regulates meiotic initiation in cultured fetal germ cells
Source: PLoS One. 2019 Nov 4;14(11):e0224628. doi: 10.1371/journal.pone.0224628 (PMC6827903; doi:10.1371/journal.pone.0224628)
Supplement: S1 Table — (PDF) [file pone.0224628.s001.pdf]

Supplemental Table S1. Antibody Information used for Western Blotting and Immunochemical Staining.

Antibody Name	Host Organism	Vendor	Catalog Number	Dilution Ratio	RRID
Anti-p38 MAPK	Rabbit	Cell Signaling Technology	9212	1:500	AB_330713
Anti-Phospho-p38 MAPK	Rabbit	Cell Signaling Technology	4511	1:500	AB_2139682
Anti-p44/42 MAPK (ERK1/2)	Rabbit	Cell Signaling Technology	4695	1:1000	AB_390779
Anti-Phospho-p44 / 42 MAPK (Thr202 / Tyr204)	Rabbit	Cell Signaling Technology	4370	1:1000	AB_2315112
Anti-Akt	Rabbit	Cell Signaling Technology	4691	1:500	AB_915783
Anti-Phospho-Akt (Ser473)	Rabbit	Cell Signaling Technology	4060	1:500	AB_2315049
Anti-SAPK/JNK	Rabbit	Cell Signaling Technology	9252	1:500	AB_2250373
Anti-Phospho-SAPK/JNK (Thr183/Tyr185)	Rabbit	Cell Signaling Technology	4668P	1:500	AB_10831195
Anti-STRA8	Rabbit	Abcam	ab49405	1:500	AB_945677
Anti-γH2A.X (Ser139)	Mouse	Abcam	ab22551	1:500	AB_447150
Anti-β-Actin	Rabbit	Cell Signaling Technology	4970	1:1000	AB_2223172
Anti-rabbit IgG HRP-linked	Goat	Cell Signaling Technology	7074	1:1000	AB_2099233
Anti-mouse IgG, Alexa Fluor 568	Goat	Thermo Fisher Scientific	A-11004	1:500	AB_2534072
Anti-rabbit IgG, Alexa Fluor 568	Goat	Thermo Fisher Scientific	A-11011	1:500	AB_143157
