# Supplementary material for: Retinoic acid-stimulated ERK1/2 pathway regulates meiotic initiation in cultured fetal germ cells
Source: PLoS One. 2019 Nov 4;14(11):e0224628. doi: 10.1371/journal.pone.0224628 (PMC6827903; doi:10.1371/journal.pone.0224628)
Supplement: S2 Table — (PDF) [file pone.0224628.s002.pdf]

Supplemental Information S2_Fig. 1A

E11.5-15.5 XX & XY germ cells

Female

	E11.5	E12.5	E13.5	E14.5	E15.5
**1**	1.45	3.05	20.09	13.02	9.18
**2**	0.55	4.03	7.36	12.95	6.21
**3**	1.00	3.54	13.26	12.90	10.73
**Ave.**	**1.00**	**3.54**	**13.57**	**12.96**	**8.71**

Male

	E11.5	E12.5	E13.5	E14.5	E15.5
**1**	0.000	0.152	0.000	0.038	1.654
**2**	1.975	0.114	0.077	0.106	0.000
**3**	1.025	0.114	0.000	0.000	0.165
**Ave.**	**1.00**	**0.13**	**0.03**	**0.05**	**0.61**
