# Supplementary material for: Retinoic acid-stimulated ERK1/2 pathway regulates meiotic initiation in cultured fetal germ cells
Source: PLoS One. 2019 Nov 4;14(11):e0224628. doi: 10.1371/journal.pone.0224628 (PMC6827903; doi:10.1371/journal.pone.0224628)
Supplement: S3 Table — (PDF) [file pone.0224628.s003.pdf]

S3 Table_Fig. 2A

E12.5 XX germ cells (1 h)

	Control	U0126 (10 µM)	U0126 (20 µM)	U0126 (50 µM)
**1**	0.83	0.36	0.26	0.19
**2**	1.17	0.42	0.29	0.22
**3**	1.00	0.39	0.21	0.15
**Ave.**	**1.00**	**0.39**	**0.25**	**0.19**
