# Supplementary material for: Retinoic acid-stimulated ERK1/2 pathway regulates meiotic initiation in cultured fetal germ cells
Source: PLoS One. 2019 Nov 4;14(11):e0224628. doi: 10.1371/journal.pone.0224628 (PMC6827903; doi:10.1371/journal.pone.0224628)
Supplement: S4 Table — (PDF) [file pone.0224628.s004.pdf]

S4 Table_Fig. 3A

E12.5 XX gonads (24 & 48h)

Stra8

	D1		D2	
	**Control**	**U0126**	**Control**	**U0126**
**1**	0.80	0.59	1.27	1.10
**2**	1.16	0.92	1.14	1.16
**3**	1.04	0.60	2.48	1.99
**Ave.**	**1.00**	**0.70**	**1.63**	**1.42**

Rec8

	D1		D2	
	**Control**	**U0126**	**Control**	**U0126**
**1**	1.18	1.00	3.73	1.91
**2**	0.36	0.18	6.82	0.09
**3**	1.45	0.82	7.64	4.18
**Ave.**	**1.00**	**0.67**	**6.06**	**2.06**

Spo11

	D1		D2	
	**Control**	**U0126**	**Control**	**U0126**
**1**	1.12	0.00	3.60	0.00
**2**	0.34	0.00	9.74	0.00
**3**	1.54	0.00	3.85	0.00
**Ave.**	**1.00**	**0.00**	**5.73**	**0.00**

Dmc1

	D1		D2	
	**Control**	**U0126**	**Control**	**U0126**
**1**	1.04	0.41	4.48	0.58
**2**	1.24	1.04	4.01	1.86
**3**	0.73	0.09	3.91	0.33
**Ave.**	**1.00**	**0.51**	**4.13**	**0.92**

Sycp3

	D1		D2	
	**Control**	**U0126**	**Control**	**U0126**
**1**	0.87	0.48	1.64	0.76
**2**	1.21	0.90	1.87	1.17
**3**	0.93	0.51	1.56	0.93
**Ave.**	**1.00**	**0.63**	**1.69**	**0.95**
