# Supplementary material for: Retinoic acid-stimulated ERK1/2 pathway regulates meiotic initiation in cultured fetal germ cells
Source: PLoS One. 2019 Nov 4;14(11):e0224628. doi: 10.1371/journal.pone.0224628 (PMC6827903; doi:10.1371/journal.pone.0224628)
Supplement: S5 Table — (PDF) [file pone.0224628.s005.pdf]

S5 Table_Fig. 3B

E12.5 XX germ cells (24 & 48h)

Stra8

	D1				D2			
	**Ctrl**	**RA**	**RA+U0126**	**U0126**	**Ctrl**	**RA**	**RA+U0126**	**U0126**
**1**	1.29	3.32	2.71	0.27	2.64	8.12	2.34	0.52
**2**	1.13	3.63	2.38	0.37	2.78	11.42	3.35	0.50
**3**	0.44	3.68	2.45	0.16	2.01	7.66	2.22	0.17
**4**	1.14	3.46	3.30	0.32	1.65	7.68	2.06	0.10
**Ave**	**1.00**	**3.52**	**2.71**	**0.28**	**2.27**	**8.72**	**2.49**	**0.32**

Rec8

	D1				D2			
	**Ctrl**	**RA**	**RA+U0126**	**U0126**	**Ctrl**	**RA**	**RA+U0126**	**U0126**
**1**	1.09	1.05	0.96	0.13	0.99	1.93	0.41	0.37
**2**	0.73	1.84	0.80	0.29	1.10	4.47	0.93	1.00
**3**	1.24	1.36	0.84	0.27	2.14	5.73	1.01	0.44
**4**	0.95	1.40	0.97	0.43	0.42	4.28	0.35	0.28
**Ave.**	**1.00**	**1.41**	**0.89**	**0.28**	**1.16**	**4.10**	**0.67**	**0.52**

Spo11

	D1				D2			
	**Ctrl**	**RA**	**RA+U0126**	**U0126**	**Ctrl**	**RA**	**RA+U0126**	**U0126**
**1**	1.23	1.96	0.00	0.00	7.62	26.14	3.60	4.22
**2**	1.06	0.95	0.37	0.01	7.64	24.95	1.88	1.94
**3**	0.16	3.83	0.91	0.00	5.50	10.07	1.72	0.12
**4**	1.55	0.04	0.01	0.06	5.47	16.57	3.97	3.02
**Ave.**	**1.00**	**1.70**	**0.32**	**0.02**	**6.56**	**19.43**	**2.79**	**2.33**
