# Supplementary material for: Retinoic acid-stimulated ERK1/2 pathway regulates meiotic initiation in cultured fetal germ cells
Source: PLoS One. 2019 Nov 4;14(11):e0224628. doi: 10.1371/journal.pone.0224628 (PMC6827903; doi:10.1371/journal.pone.0224628)
Supplement: S6 Table — (PDF) [file pone.0224628.s006.pdf]

S6 Table_Fig. 4B

E12.5 XX germ cells (24h)

1st	Hoechst	STRA8-positive	Stra8-negative	% of STRA8-positive cells	% of STRA8-negative cells
**Control**	102	56	46	54.9	45.1
**RA**	105	59	46	56.2	43.8
**RA+U0126**	87	33	54	37.9	62.1
**U0126**	28	5	23	17.9	82.1

2nd	Hoechst	STRA8-positive	Stra8-negative	% of STRA8-positive cells	% of STRA8-negative cells
**Control**	100	58	42	58.0	42.0
**RA**	98	60	38	61.2	38.8
**RA+U0126**	86	30	56	34.9	65.1
**U0126**	80	12	68	15.0	85.0

3rd	Hoechst	STRA8-positive	Stra8-negative	% of STRA8-positive cells	% of STRA8-negative cells
**Control**	90	50	40	55.6	44.4
**RA**	85	50	35	58.8	41.2
**RA+U0126**	80	28	52	35.0	65.0
**U0126**	85	13	72	15.3	84.7

Total

	Hoechst	STRA8-positive	Stra8-negative	% of STRA8-positive cells	% of STRA8-negative cells
**Control**	292	164	128	56.2	43.8
**RA**	288	169	119	58.7	41.3
**RA+U0126**	253	91	162	36.0	64.0
**U0126**	193	30	163	15.5	84.5
