# Supplementary material for: Retinoic acid-stimulated ERK1/2 pathway regulates meiotic initiation in cultured fetal germ cells
Source: PLoS One. 2019 Nov 4;14(11):e0224628. doi: 10.1371/journal.pone.0224628 (PMC6827903; doi:10.1371/journal.pone.0224628)
Supplement: S7 Table — (PDF) [file pone.0224628.s007.pdf]

S7 Table_Fig. 4C

E12.5 XX germ cells (24h)

Cell #	Control	RA	RA+U0126	U0126		Control	RA	RA+U0126	U0126		Control	RA	RA+U0126	U0126
**1**	76	74	68	24		41	77	42	10		26	82	17	14
**2**	56	81	56	19		33	81	13	25		28	86	14	23
**3**	39	77	33	22		30	76	28	15		26	90	16	28
**4**	43	78	32	21		32	72	23	0		27	87	30	0
**5**	52	73	38	18		36	72	27	0		26	91	18	0
**6**	62	64	66	0		26	93	16	0		45	85	16	0
**7**	77	40	35	0		60	88	13	0		52	70	44	0
**8**	57	48	40	0		28	74	63	0		42	61	17	0
**9**	68	49	36	0		36	55	27	0		26	57	14	0
**10**	54	77	64	0		29	57	43	0		27	56	27	0
**11**	36	49	36			28	91	43			46	56	42	
**12**	68	60	65			28	84	14			29	59	13	
**13**	62	68	66			26	88	40			31	58	13	
**14**	58	64	52			26	57	13			32	58	13	
**15**	68	66	38			26	57	13			27	60	15	
**16**	53	74	35			30	89	13			30	59	13	
**17**	69	76	43			26	85	14			33	58	13	
**18**	46	70	31			26	55	13			26	79	26	
**19**	65	81	30			27	58	13			26	55	13	
**20**	64	78	39			26	57	52			26	59	13	
**21**	68	82	39			43	55	68			26	73	19	
**22**	67	72	55			27	73	15			27	56	26	
**23**	59	82	27			26	58	19			26	55	17	
**24**	26	82	44			26	61	18			27	60	44	
**25**	42	42	17			36	58	14			26	80	14	
**26**	42	40	15			26	55	14			28	87	15	
**27**	26	44	31			27	62	30			41	86		
**28**	39	53				26	57				27	58		
**29**	33	70				27	56				49	58		
**30**	39	70				26	55				26	55		
**31**	26	47				26	55				26	62		
**32**	26	52				27	91				26	74		
**33**	27	72				26	87				32	56		
**34**	27	76				39	63				26	57		
**35**	26	76				26	73				26	55		
**36**	46	40				42	57				26	85		
**37**	26	66				26	86				26	55		
**38**	26	56				43	76				26	76		
**39**	26	82				26	73				26	72		
**40**	32	60				26	76				26	59		
**41**	50	67				26	57				41	60		
**42**	26	55				26	82				26	75		
**43**	42					29					27			
**44**	26					26					35			
**45**	42					34					45			
**46**	27					26					28			
**47**	26					26					48			
**48**	36					26					26			
**49**	26					54					45			
**50**	29					27					26			
**51**	26					26					26			
**52**	26					28					46			
**53**	26					33					32			
**54**	46					26					27			
**55**	26					27					26			
**56**	35					39					26			
**57**	26					27					26			
**Ave.**	**42.86**	**65.07**	**41.89**	**10.40**		**30.23**	**69.81**	**25.96**	**5.00**		**30.81**	**67.14**	**20.08**	**6.50**
