# Supplementary material for: Retinoic acid-stimulated ERK1/2 pathway regulates meiotic initiation in cultured fetal germ cells
Source: PLoS One. 2019 Nov 4;14(11):e0224628. doi: 10.1371/journal.pone.0224628 (PMC6827903; doi:10.1371/journal.pone.0224628)
Supplement: S8 Table — (PDF) [file pone.0224628.s008.pdf]

S8 Table_Fig. 4E

E12.5 XX germ cells (24h)

1st	Hoechst	γH2AX-positive	γH2AX-negative	% of γH2AX-positive cells
Control	52	36	16	69.2
RA	39	28	11	71.8
RA+U0126	62	18	44	29.0
U0126	30	4	26	13.3

2nd	Hoechst	γH2AX-positive	γH2AX-negative	% of γH2AX-positive cells
Control	44	24	20	54.5
RA	39	24	15	61.5
RA+U0126	45	7	38	15.6
U0126	32	3	29	9.4

Total

	Hoechst	γH2AX-positive	γH2AX-negative	% of γH2AX-positive cells
Control	96	60	36	62.5
RA	78	52	26	66.7
RA+U0126	107	25	82	23.4
U0126	62	7	55	11.3

% of γH2AX-negative cells
30.8
28.2
71.0
86.7

% of γH2AX-negative cells
45.5
38.5
84.4
90.6

% of γH2AX-negative cells
37.5
33.3
76.6
88.7
