# Supplementary material for: Retinoic acid-stimulated ERK1/2 pathway regulates meiotic initiation in cultured fetal germ cells
Source: PLoS One. 2019 Nov 4;14(11):e0224628. doi: 10.1371/journal.pone.0224628 (PMC6827903; doi:10.1371/journal.pone.0224628)
Supplement: S9 Table — (PDF) [file pone.0224628.s009.pdf]

S9 Table_Fig. 5C
E13.5 XY germ cells (48 & 72h)

Stra8

	D2				D3			
	Ctrl	RA	RA+U0126	U0126	Ctrl	RA	RA+U0126	U0126
1	2.95	4.87	4.39	0.00	0.50	6.55	1.10	0.01
2	0.62	4.86	3.25	0.11	0.71	7.73	2.25	0.04
3	0.73	5.64	3.41	0.22	0.39	2.93	0.30	0.00
4	0.36	9.91	3.77	0.00				
5	0.67	5.38	1.21	0.03				
6	0.67	1.64	0.56	0.00				
**Ave.**	**1.00**	**5.38**	**2.76**	**0.06**	**0.54**	**5.74**	**1.22**	**0.02**

Rec8

	D2				D3			
	Ctrl	RA	RA+U0126	U0126	Ctrl	RA	RA+U0126	U0126
1	1.80	4.61	3.98	1.02	0.98	5.01	2.13	2.40
2	0.74	1.76	1.92	0.81	0.51	1.86	0.85	0.52
3	0.46	1.22	0.21	0.23	0.77	1.75	0.29	0.31
**Ave.**	**1.00**	**2.53**	**2.04**	**0.69**	**0.75**	**2.88**	**1.09**	**1.07**

Spo11

	D2				D3			
	Ctrl	RA	RA+U0126	U0126	Ctrl	RA	RA+U0126	U0126
1	0.27	4.26	7.22	1.69	1.92	28.51	5.91	0.31
2	1.89	16.10	11.04	1.42	11.86	24.87	0.00	4.97
3	0.84	1.76	3.41	1.83	18.11	38.33	0.00	0.93
**Ave.**	**1.00**	**7.37**	**7.22**	**1.65**	**10.63**	**30.57**	**1.97**	**2.07**

Dmc1

	D2				D3			
	Ctrl	RA	RA+U0126	U0126	Ctrl	RA	RA+U0126	U0126
1	0.85	0.80	0.00	0.00	1.95	4.47	0.00	0.15
2	1.33	2.81	2.31	0.69	1.97	9.37	2.10	0.25
3	0.82	0.76	1.99	0.00	1.65	7.79	1.00	0.35
**Ave.**	**1.00**	**1.46**	**1.43**	**0.23**	**1.86**	**7.21**	**1.03**	**0.25**

Sycp3

	D2				D3			
	Ctrl	RA	RA+U0126	U0126	Ctrl	RA	RA+U0126	U0126
1	0.66	1.06	1.32	0.77	0.71	1.89	0.61	0.53
2	1.46	1.28	0.67	0.73	0.85	2.44	0.69	0.49
3	0.88	1.78	0.06	0.36	0.93	2.56	1.04	0.12
**Ave.**	**1.00**	**1.37**	**0.68**	**0.62**	**0.83**	**2.30**	**0.78**	**0.38**
