# Supplementary material for: Retinoic acid-stimulated ERK1/2 pathway regulates meiotic initiation in cultured fetal germ cells
Source: PLoS One. 2019 Nov 4;14(11):e0224628. doi: 10.1371/journal.pone.0224628 (PMC6827903; doi:10.1371/journal.pone.0224628)
Supplement: S10 Table — (PDF) [file pone.0224628.s010.pdf]

Supplemental Information S10_Fig. 6B

E13.5 XY PGCs (48 h)

1st	Hoechst	STRA8-positive	STRA8-negative	% of STRA8-positive cells	% of STRA8-negative cells
**Control**	54	0	54	0.0	100.0
**RA**	70	53	17	75.7	24.3
**RA+U0126**	94	16	78	17.0	83.0
**U0126**	85	0	85	0.0	100.0

2nd	Hoechst	STRA8-positive	STRA8-negative	% of STRA8-positive cells	% of STRA8-negative cells
**Control**	108	0	108	0.0	100.0
**RA**	142	106	36	74.6	25.4
**RA+U0126**	180	32	148	17.8	82.2
**U0126**	162	5	157	3.1	96.9

3rd	Hoechst	STRA8-positive	STRA8-negative	% of STRA8-positive cells	% of STRA8-negative cells
**Control**	111	0	111	0.0	100.0
**RA**	209	151	58	72.2	27.8
**RA+U0126**	176	36	140	20.5	79.5
**U0126**	157	6	151	3.8	96.2

Total

	Hoechst	STRA8-positive	STRA8-negative	% of STRA8-positive cells	% of STRA8-negative cells
**Control**	273	0	273	0.0	100.0
**RA**	421	310	111	73.6	26.4
**RA+U0126**	450	84	366	18.7	81.3
**U0126**	404	11	393	2.7	97.3
