# Supplementary material for: Retinoic acid-stimulated ERK1/2 pathway regulates meiotic initiation in cultured fetal germ cells
Source: PLoS One. 2019 Nov 4;14(11):e0224628. doi: 10.1371/journal.pone.0224628 (PMC6827903; doi:10.1371/journal.pone.0224628)
Supplement: S11 Table — (PDF) [file pone.0224628.s011.pdf]

# Supplemental Information S11_Fig. 6D

E13.5 XY PGCs (48 h)

| 1st | Hoechst | γH2AX-positive | γH2AX-negative | % of γH2AX-positive cells | % of γH2AX-negative cells |
|---|---|---|---|---|---|
| **Control** | 75 | 2 | 73 | 2.7 | 97.3 |
| **RA** | 40 | 15 | 25 | 37.5 | 62.5 |
| **RA+U0126** | 130 | 6 | 124 | 4.6 | 95.4 |
| **U0126** | 74 | 4 | 70 | 5.4 | 94.6 |

| 2nd | Hoechst | γH2AX-positive | γH2AX-negative | % of γH2AX-positive cells | % of γH2AX-negative cells |
|---|---|---|---|---|---|
| **Control** | 176 | 3 | 173 | 1.7 | 98.3 |
| **RA** | 31 | 9 | 22 | 29.0 | 71.0 |
| **RA+U0126** | 80 | 4 | 76 | 5.0 | 95.0 |
| **U0126** | 72 | 2 | 70 | 2.8 | 97.2 |

| 3rd | Hoechst | γH2AX-positive | γH2AX-negative | % of γH2AX-positive cells | % of γH2AX-negative cells |
|---|---|---|---|---|---|
| **Control** | 86 | 2 | 84 | 2.3 | 97.7 |
| **RA** | 98 | 42 | 56 | 42.9 | 57.1 |
| **RA+U0126** | 124 | 9 | 115 | 7.3 | 92.7 |
| **U0126** | 143 | 3 | 140 | 2.1 | 97.9 |

**Total**

| | Hoechst | γH2AX-positive | γH2AX-negative | % of γH2AX-positive cells | % of γH2AX-negative cells |
|---|---|---|---|---|---|
| **Control** | 337 | 7 | 330 | 2.1 | 97.9 |
| **RA** | 169 | 66 | 103 | 39.1 | 60.9 |
| **RA+U0126** | 334 | 19 | 315 | 5.7 | 94.3 |
| **U0126** | 289 | 9 | 280 | 3.1 | 96.9 |
