# Supplementary material for: Retinoic acid-stimulated ERK1/2 pathway regulates meiotic initiation in cultured fetal germ cells
Source: PLoS One. 2019 Nov 4;14(11):e0224628. doi: 10.1371/journal.pone.0224628 (PMC6827903; doi:10.1371/journal.pone.0224628)
Supplement: S12 Table — (PDF) [file pone.0224628.s012.pdf]

S12 Table_S2 Fig. A

E12.5 XX gonads (24 & 48h)

Mvh

	D1		D2	
	**Control**	**U0126**	**Control**	**U0126**
**1**	1.12	1.22	1.39	1.12
**2**	1.01	0.89	1.06	1.45
**3**	0.87	1.26	2.45	1.57
**Ave.**	1.00	1.12	1.63	1.38
