# Supplementary material for: Retinoic acid-stimulated ERK1/2 pathway regulates meiotic initiation in cultured fetal germ cells
Source: PLoS One. 2019 Nov 4;14(11):e0224628. doi: 10.1371/journal.pone.0224628 (PMC6827903; doi:10.1371/journal.pone.0224628)
Supplement: S13 Table — (PDF) [file pone.0224628.s013.pdf]

S13 Table_S2 Fig. B

E12.5 XX germ cells (24 & 48h)

Mvh

	D1				D2			
	Ctrl	RA	RA+U0126	U0126	Ctrl	RA	RA+U0126	U0126
1	0.33	1.25	0.83	0.72	2.41	2.12	1.06	0.81
2	1.63	1.22	1.02	0.81	1.12	0.79	0.65	0.41
3	0.08	0.32		0.71				
4	1.96	0.81	0.93	0.84	2.66	2.85	0.80	0.89
**Ave**	**1.00**	**0.90**	**0.93**	**0.77**	**2.06**	**1.92**	**0.84**	**0.70**
