# Supplementary material for: Retinoic acid-stimulated ERK1/2 pathway regulates meiotic initiation in cultured fetal germ cells
Source: PLoS One. 2019 Nov 4;14(11):e0224628. doi: 10.1371/journal.pone.0224628 (PMC6827903; doi:10.1371/journal.pone.0224628)
Supplement: S14 Table — (PDF) [file pone.0224628.s014.pdf]

S14 Table_S3 Fig.
E13.5 XY germ cells (48 & 72h)

Mvh

	D2				D3			
	**Ctrl**	**RA**	**RA+U0126**	**U0126**	**Ctrl**	**RA**	**RA+U0126**	**U0126**
**1**	0.42	0.77	1.19	0.83	0.94	1.28	0.86	0.65
**2**	0.76	0.93	0.83	0.91	0.95	1.45	0.65	0.61
**3**	2.28	1.41	1.55	1.22				
**4**	0.55	1.12	1.34	0.85	1.64	2.10	1.03	0.78
**Ave**	**1.00**	**1.06**	**1.23**	**0.95**	**1.18**	**1.61**	**0.85**	**0.68**
