# Supplementary material for: Retinoic acid-stimulated ERK1/2 pathway regulates meiotic initiation in cultured fetal germ cells
Source: PLoS One. 2019 Nov 4;14(11):e0224628. doi: 10.1371/journal.pone.0224628 (PMC6827903; doi:10.1371/journal.pone.0224628)
Supplement: S15 Table — For signal transduction pathways, the β-ACTIN-normalized phospho/total protein ratio was set as the O.D. value of each phosphorylated protein. The O.D. value of STRA8 was determined by normalizing to the β-ACTIN. The data represent the mean ± SD of two independent WB experiments. (PDF) [file pone.0224628.s015.pdf]

S15 Table_O.D. for Westren blot

Fig. 1B

p-ERK1/2

	0h	0.5h	1h	2h
1	2828	4404	7835	22888
2	1828	3458	6855	19288
**Ave**	**2328**	**3931**	**7345**	**21088**
**SD**	**707**	**669**	**693**	**2546**

p-P38

	0h	0.5h	1h	2h
1	24738	12968	13385	18268
2	10738	6968	7385	9268
**Ave**	**17738**	**9968**	**10385**	**13768**
**SD**	**9899**	**4243**	**4243**	**6364**

Fig. 2B

STRA8

	Control	RA	RA+U0126	U0126
1	910	30966	6242	859
2	752	25476	3522	255
**Ave**	**831**	**28221**	**4882**	**557**
**SD**	**112**	**3882**	**1923**	**427**

p-ERK1/2

	Control	RA	RA+U0126	U0126
1	1804	31479	2690	1108
2	2914	24529	2050	568
**Ave**	**2359**	**28004**	**2370**	**838**
**SD**	**785**	**4914**	**453**	**382**

Fig. 5A

STRA8

	0h	12h	24h	48h
1	3911	26409	48064	134453
2	1452	2253	8523	94285
**Ave**	**2682**	**14331**	**28294**	**114369**
**SD**	**1739**	**17081**	**27960**	**28403**

p-ERK1/2

	0h	12h	24h	48h
1	7219	18636	31181	121719
2	5222	13020	22332	62355
**Ave**	**6221**	**15828**	**26757**	**92037**
**SD**	**1412**	**3971**	**6257**	**41977**

Fig. 5B

STRA8

	Control	RA	RA+U0126	U0126
1	0	32777	2751	0
2	0	12777	751	0
**Ave**	**0**	**22777**	**1751**	**0**
**SD**	**0**	**14142**	**1414**	**0**

p-ERK1/2

	Control	RA	RA+U0126	U0126
1	0	37024	0	0
2	0	17454	0	0
**Ave**	**0**	**27239**	**0**	**0**
**SD**	**0**	**13838**	**0**	**0**

S1 Fig.

p-ERK1/2

	0 µM	10 µM	20 µM	50 µM
1	29287	23427	22428	6006
