# Supplementary figures and images for: Retinoic acid-stimulated ERK1/2 pathway regulates meiotic initiation in cultured fetal germ cells
Source: PLoS One. 2019 Nov 4;14(11):e0224628. doi: 10.1371/journal.pone.0224628 (PMC6827903; doi:10.1371/journal.pone.0224628)

**S1 Fig.**

**E12.5 XX germ cells (1h)**

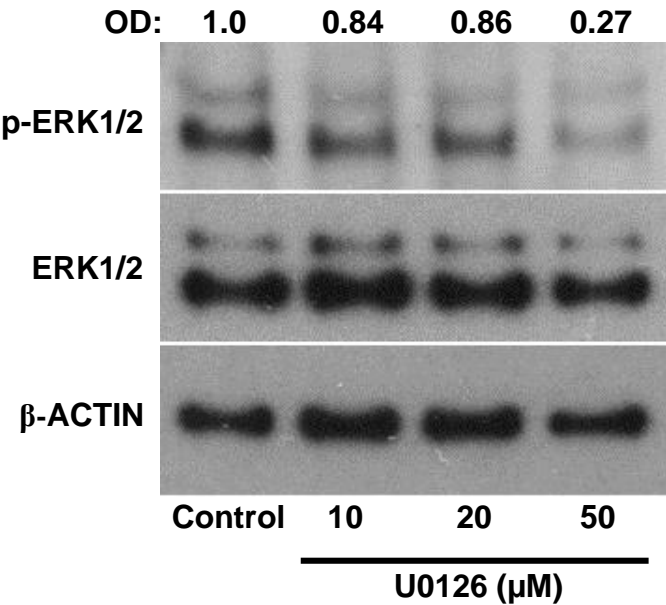

Supplement: S1 Fig — To evaluate the effect of U0126 on the ERK1/2 phosphorylation, XX germ cells at E12.5 were cultured with a MEK inhibitor (U0126) at different concentrations (0, 10, 20, and 50 μM) for 1h. After culture, germ cells were subjected to Western blotting to quantify the ERK1/2 phosphorylation. The levels of protein bands were quantified by densitometry and represented on the top of each band as numerical values that calculated relative to the control set as 1.0. (PDF) [file pone.0224628.s016.pdf]

**S2 Fig. A**

**E12.5 XX germ cells in cultured gonads (24 & 48h)**

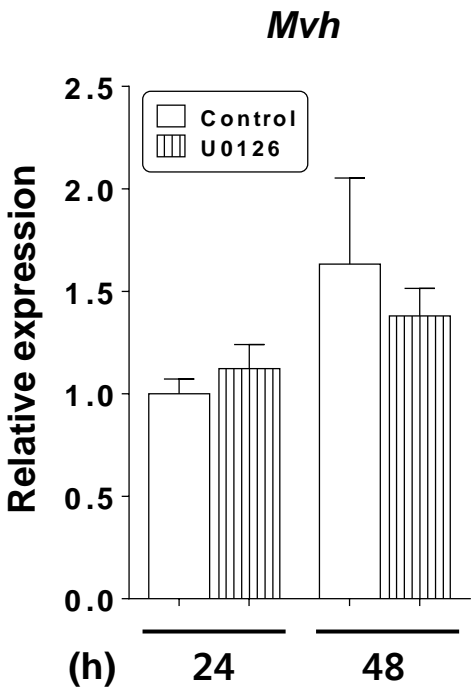

**B**

**E12.5 XX germ cells (24 & 48h)**

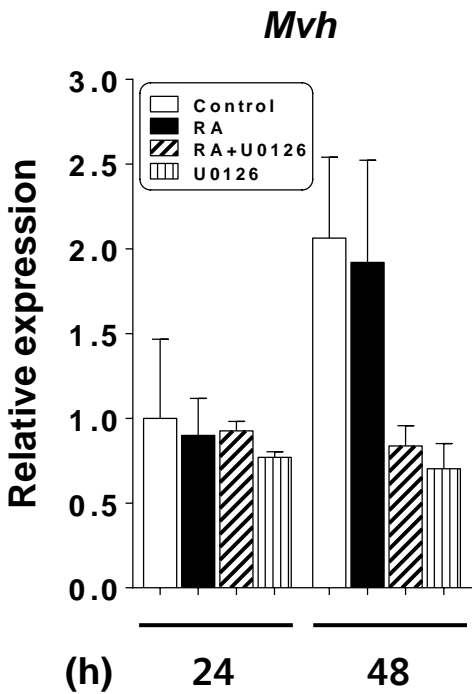

Supplement: S2 Fig — (A) Female gonads at E12.5 were cultured with 50 μM U0126 (U0126) or without treatment (control) for 24 or 48h. After culture, germ cells were collected to analysis the transcript expressions of mouse Vasa homolog (Mvh). Results were normalized to the β -actin transcript expression. All expression values were calculated relative to control levels set at 1.0. Data represent the mean ± SEM (n = 3). (B) Sorted XX germ cells at E12.5 were cultured under four different conditions (control, RA, RA+U0126, U0126) for 24 and 48h. After culture, the cells were subjected to qPCR analysis for Mvh. Each gene expressions were normalized to the β-actin expression. All expression values were calculated relative to control levels set at 1.0. Data represent the mean ± SEM (n = 4). (PDF) [file pone.0224628.s017.pdf]

**S3 Fig.**

**E13.5 XY germ cells (48 & 72h)**

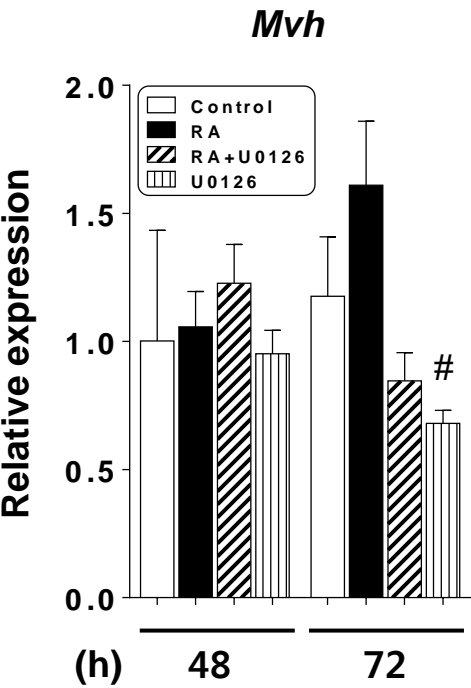

Supplement: S3 Fig — Isolated E13.5 XY germ cells were cultured under four different conditions (control, RA, RA+U0126, U0126) for 48 and 72h. After culture, the cells were subjected to qPCR analysis to determine the transcript levels of Mvh. The expression levels were normalized to β -actin mRNA expression. All expression values were calculated relative to control levels set at 1.0. Data represent the mean ± SEM (n = 3–4). # p < 0.05 vs. RA. (PDF) [file pone.0224628.s018.pdf]
